# Supplementary material for: Constraining climate sensitivity and continental versus seafloor weathering using an inverse geological carbon cycle model
Source: Nat Commun. 2017 May 22;8:15423. doi: 10.1038/ncomms15423 (PMC5458154; doi:10.1038/ncomms15423)
Supplement: Supplementary Information — Supplementary Figures, Supplementary Notes, Supplementary Methods and Supplementary References [file ncomms15423-s1.pdf]

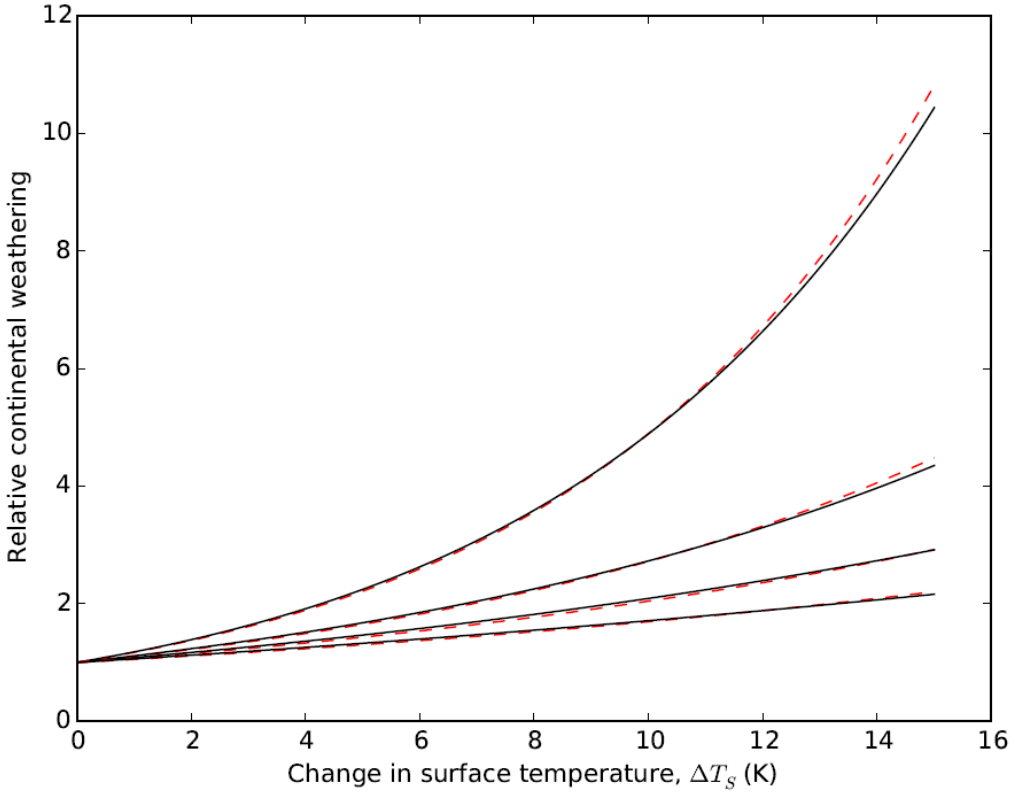

Supplementary Figure 1: This figure justifies the omission of a linear runoff dependence in our continental weathering function. The four black lines are defined by the function relative weathering = $\exp(\Delta T_S / T_e)(1 + 0.04 \times \Delta T_S)$, where the lines have effective temperatures of T_e = 8, 15, 25, and 50 K. The four red dashed lines are exponentials, relative weathering = $\exp(\Delta T_S / T_e)$, with effective temperatures chosen to fit the black lines (T_e = 6.3, 10, 14, 19 K). We see that for the range of temperature variations we are considering, any weathering function with a runoff term can be fitted with a single exponential, and hence it is appropriate to model the overall temperature dependence of continental weathering using an exponential.

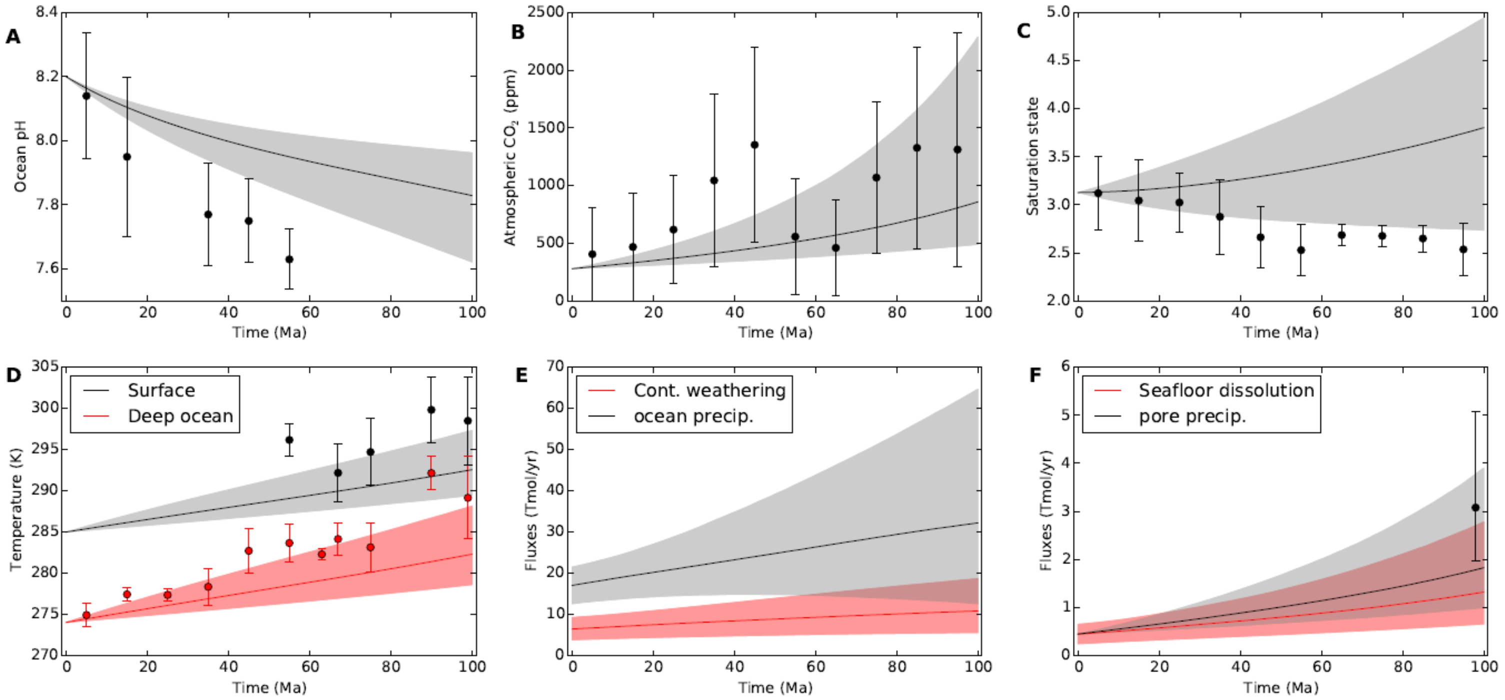

Supplementary Figure 2: Selected model outputs and geochemical proxy data for a conventional temperature sensitivity range for continental weathering ($T_e = 5 \text{ to } 15\ \mathrm{K}$) and a 40-60% change in continental weatherability over the last 100 Ma (*W*=-0.6 to -0.4). Grey and red shaded regions represent the model output 90% confidence obtained from 10,000 forward model runs using the parameter ranges described in Table 1. The grey and red solid lines are the median model outputs. Black and red dots represent binned geochemical proxy data, and error bars denote the range of binned proxy estimates (see main text for references and explanation). Here, the model envelopes marginally encompass the proxy data. The upper end of the temperature and seafloor envelopes fit proxies, pCO_2 is an excellent fit, and the saturation state and pH proxies are slightly outside the envelope.

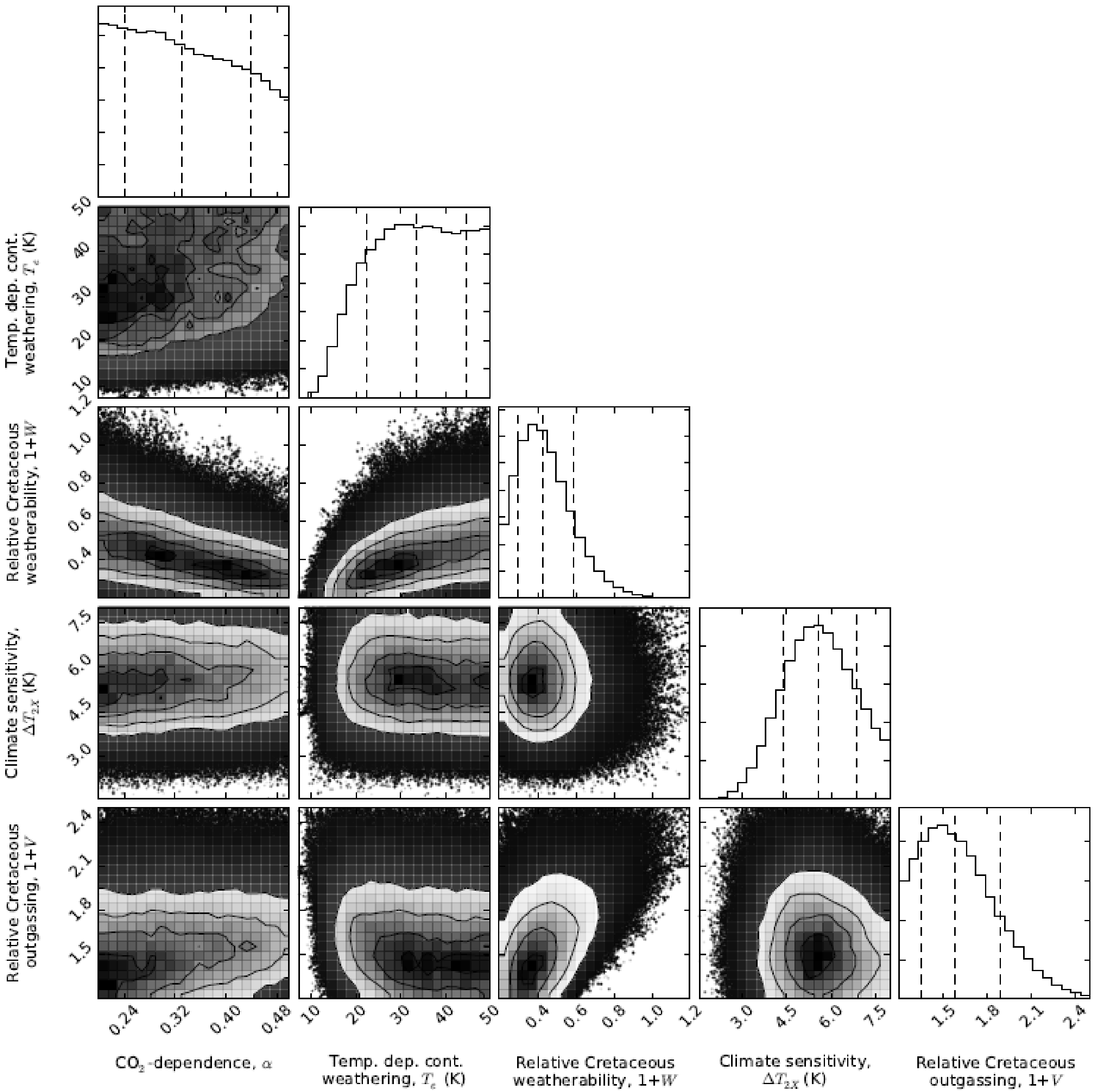

Supplementary Figure 3: Posterior probability distributions for selected carbon cycle variables from Bayesian MCMC analysis. The outer diagonal elements are the marginal distributions, identical to Fig. 6 in the main text (Column 1, nominal model), where dotted lines denote median values and 1σ error bars. The off-diagonal elements are joint probability distributions, which show how each pair of variables co-varies. The joint distributions show degeneracies that could be resolved with better data. For example, there is a positive correlation between Cretaceous weatherability and Cretaceous outgassing; if outgassing at 100 Ma was high, then the weatherability change was necessarily modest, and vice versa. This degeneracy highlights uncertainty surrounding Cretaceous climate: whether high pCO_2 levels were caused by enhanced outgassing or reduced CO_2 sinks. If either variable could be constrained by data, then the retrieval would be more informative.

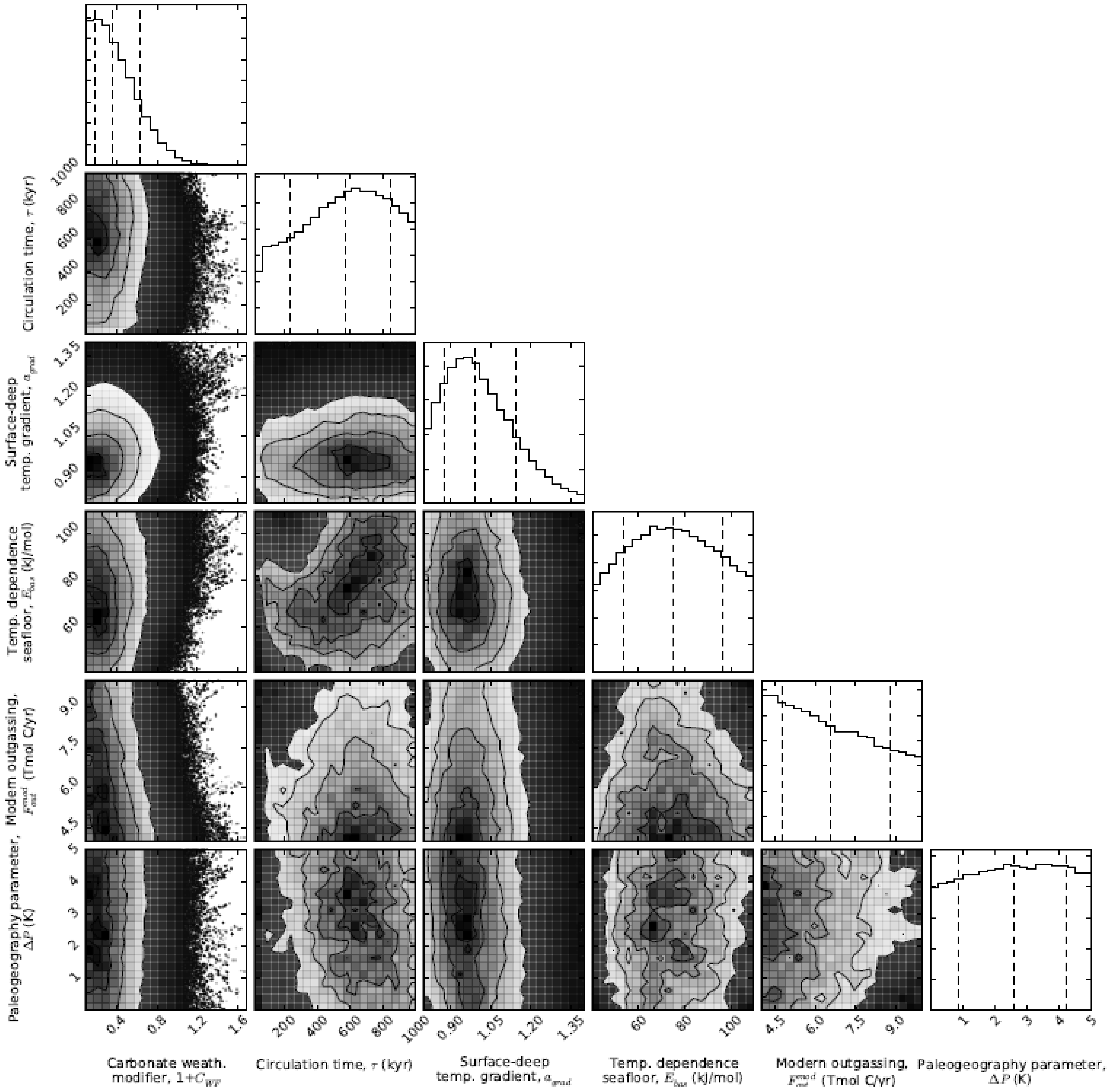

Supplementary Figure 4: Posterior distributions for variables that were omitted from Fig. 6. The outer diagonal elements are the marginal distributions, and the off-diagonal elements are joint probability distributions, which show how each pair of variables co-varies. Dotted lines represent the median value with 1σ error bars. Marginal distributions place tentative constraints on pore-space circulation time, the relationship between deep ocean and surface temperatures, and the effective activation energy for seafloor dissolution.

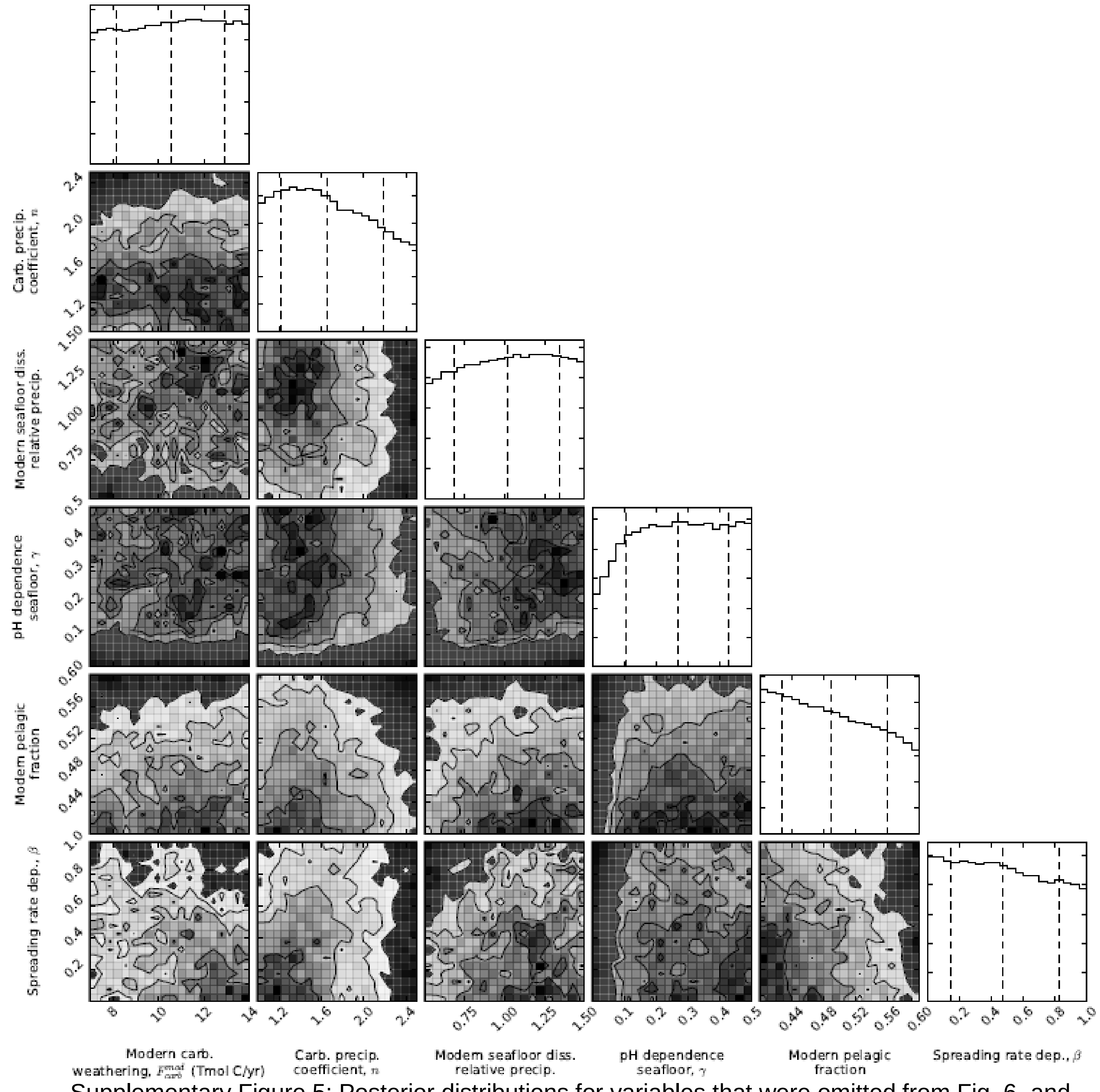

Supplementary Figure 5: Posterior distributions for variables that were omitted from Fig. 6, and supplementary figures 3 and 4. The outer diagonal elements are the marginal distributions, and the off-diagonal elements are joint probability distributions, which show how each pair of variables co-varies. Dotted lines represent the median value with 1σ error bars. Here the marginal distributions are relatively flat, indicating that these variables are not constrained by the data.

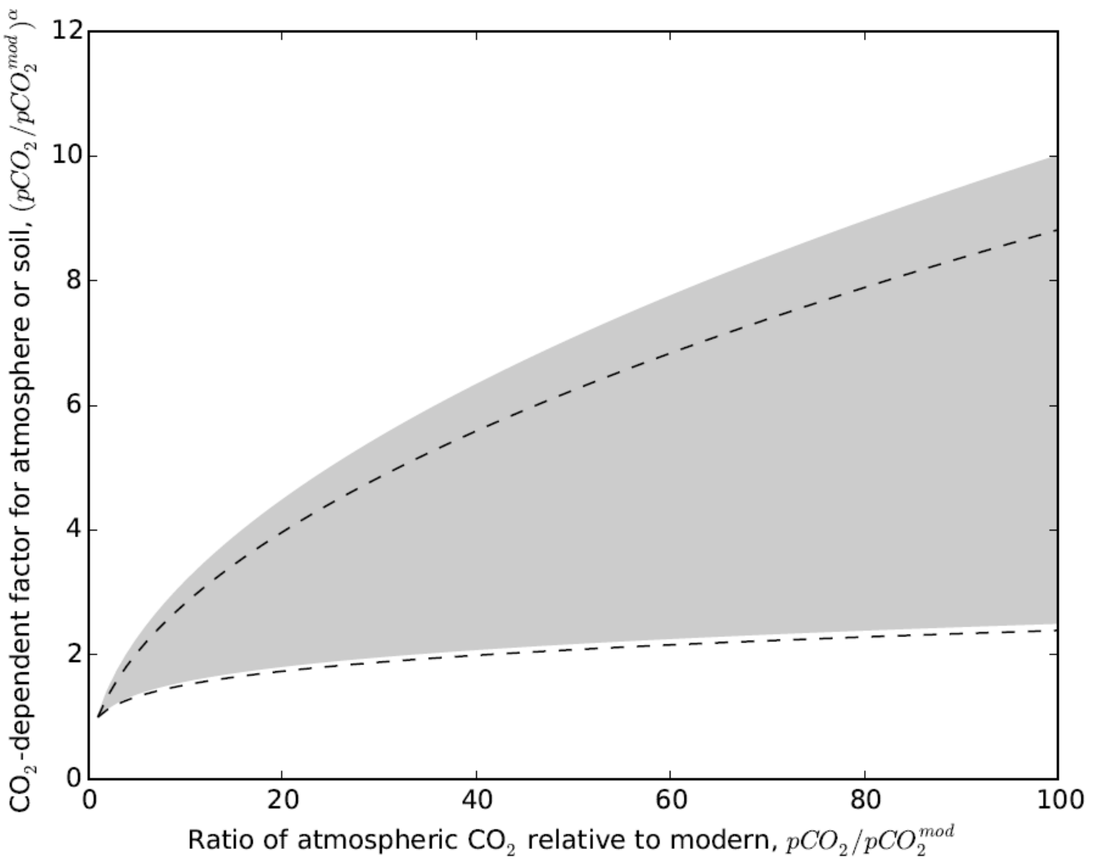

Supplementary Figure 6: Comparison of $\left(pCO_2/pCO_2^{mod}\right)^{\alpha}$ factor in equation (2) using pCO_2 values for the atmosphere or soil. The grey shaded region is the range of $\left(pCO_2/pCO_2^{mod}\right)^{\alpha}$ assumed in our model, where α =0.2-0.5. The black dotted lines bound the range of curves if soil pCO_2 is used instead, adopting the model of Volk[1] to link soil and atmospheric pCO_2, and assuming a maximum biosphere productivity of 4 times the modern productivity. We see that for our purposes it is valid to use atmospheric CO_2 in equation (2) rather than soil pCO_2.

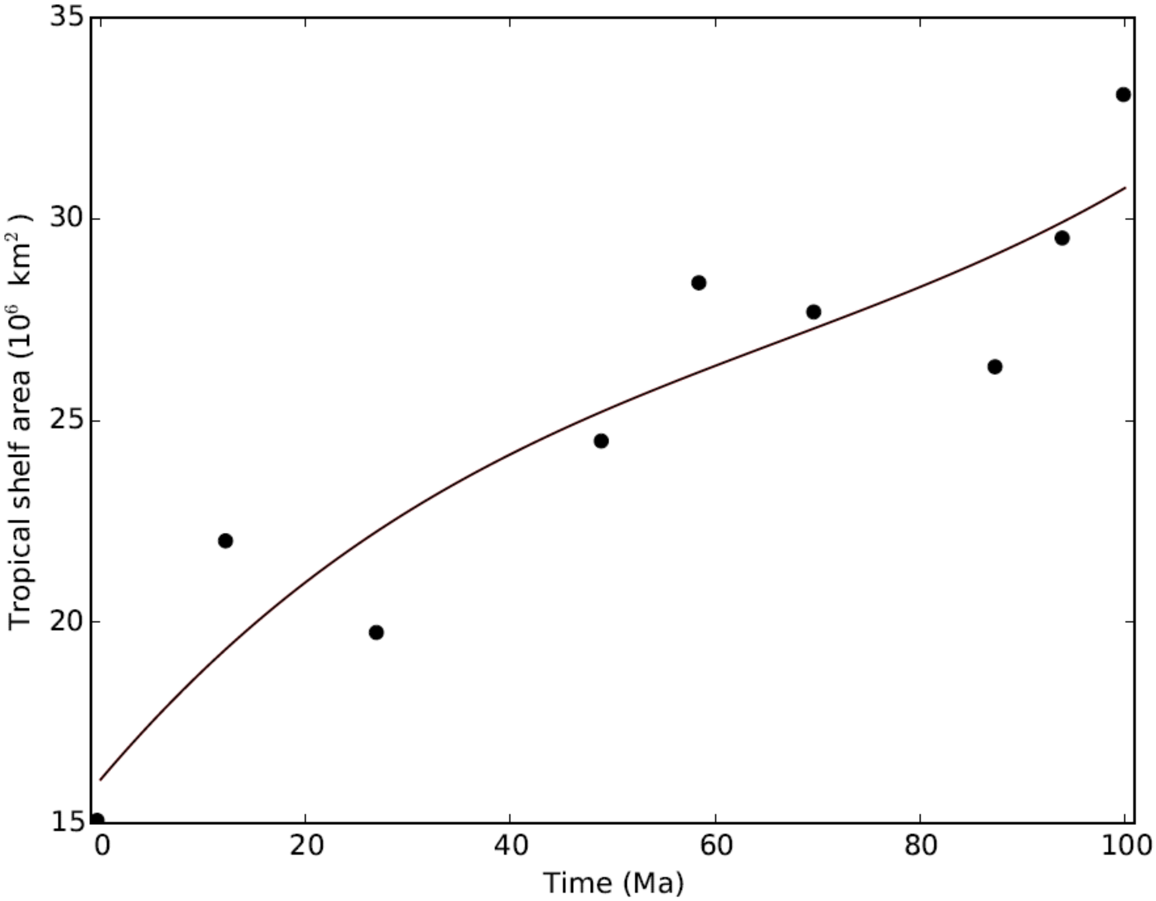

Supplementary Figure 7: Tropical shelf area estimates from Walker *et al.*[2] (circles) with the polynomial fit used in this study (solid line). The vast majority of shelf carbonates precipitate in tropical latitudes (0-30 degrees), and so we use reconstructed tropical shelf area to represent A_{shelf}. The fit is given by $A_{\mathrm{shelf}} = 1.54\times10^{-23}t^3 - 3.072\times10^{-15}t^2 + 2.9997\times10^{-7}t + 16.089$

Here, t is in years since the present, and A_{shelf} is in 10^6 km^2.

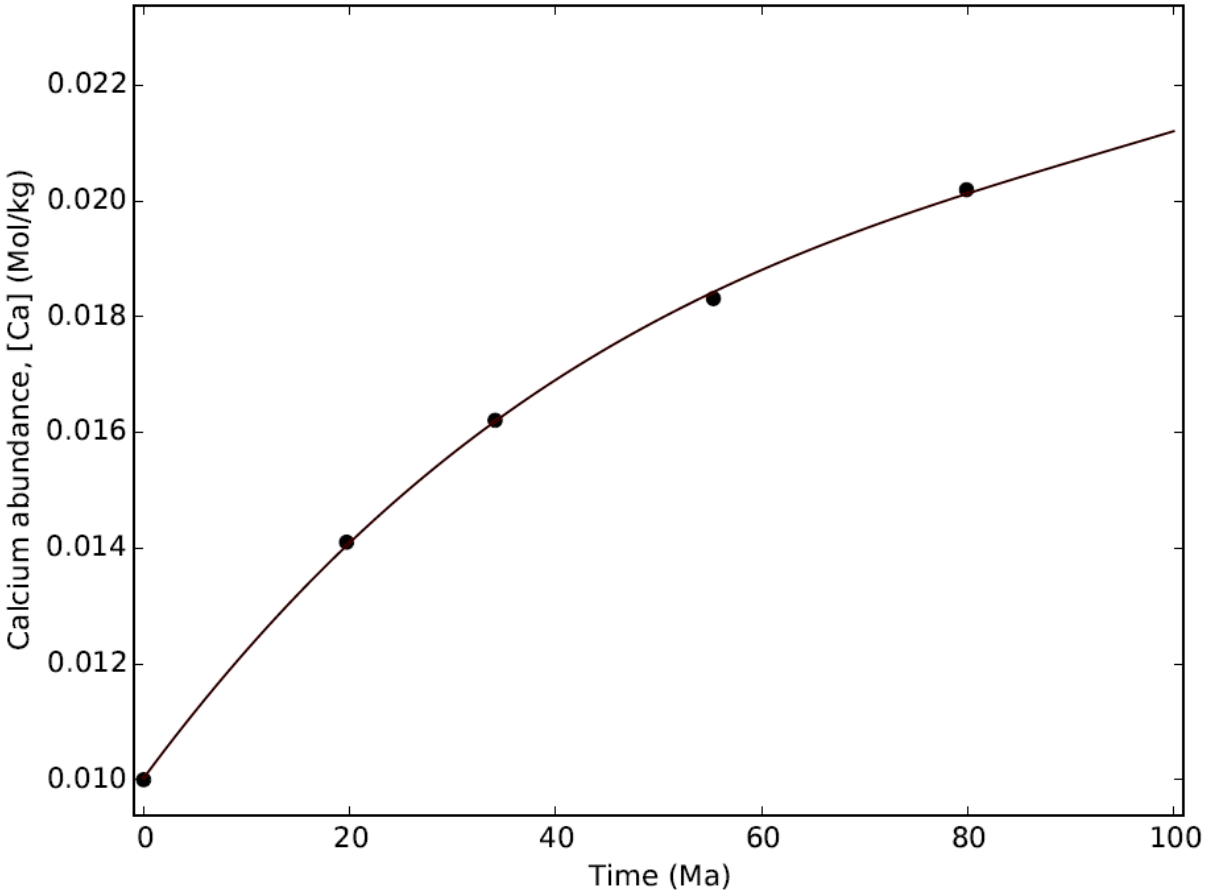

Supplementary Figure 8: Dissolved calcium ion abundance estimates from Tyrrell and Zeebe[3] for the last 100 Ma (circles) with the polynomial fit used in this study (solid line):

$$\left[Ca^{2+}\right] = 7.00658\times10^{-27}t^3 - 1.9847\times10^{-18}t^2 + 2.4016\times10^{-10}t + 0.0100278$$

Here, t is in years since the present, and $[Ca^{2+}]$ is in mol kg^{-1}. This expression was used for the calcium abundance in both the ocean and the pore-space.

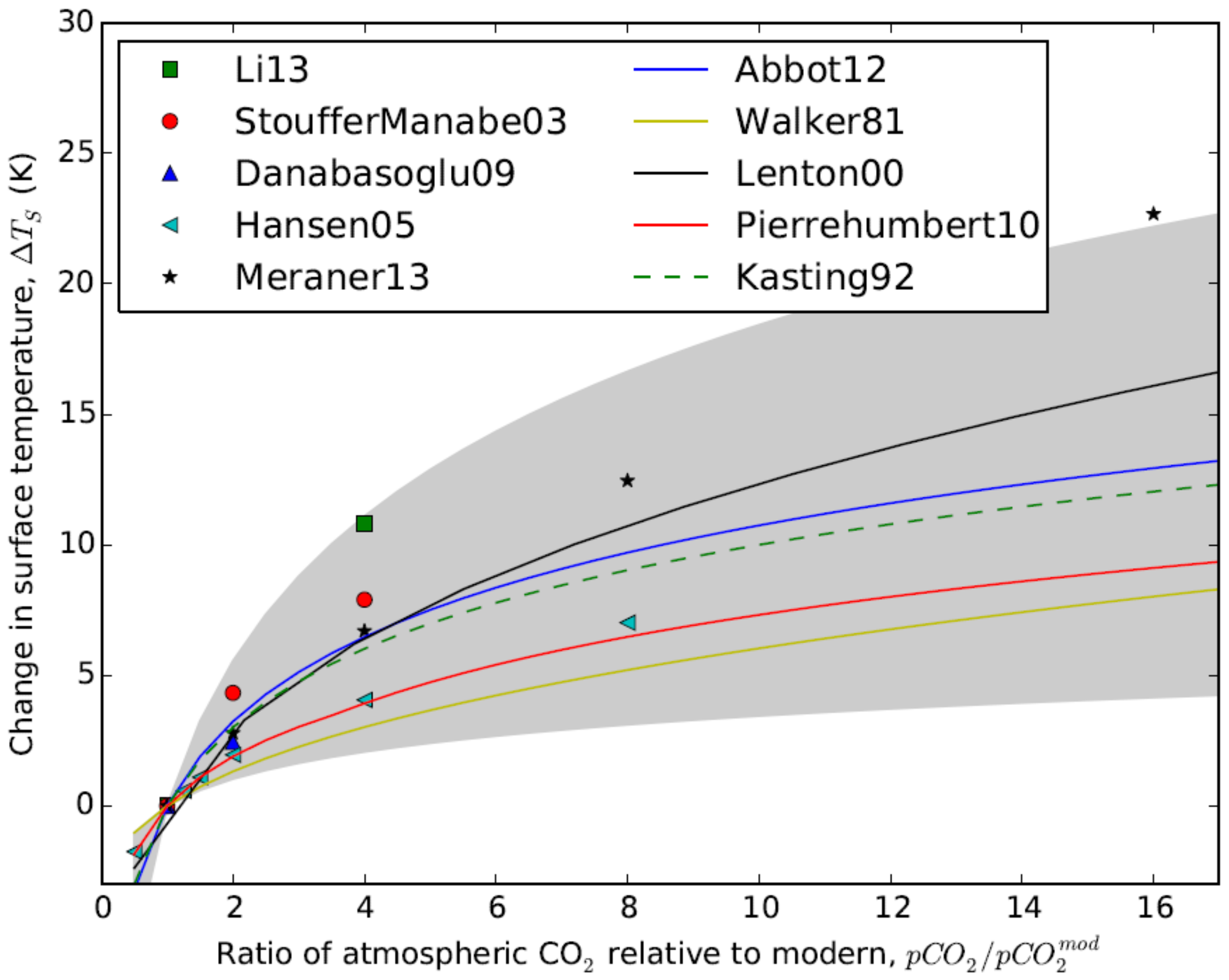

Supplementary Figure 9: A selection of GCM outputs (colored symbols) and simple climate models (lines) from the literature. The grey shaded region is the range of climate parameterizations considered in our model, equation (9), where the climate sensitivity, ΔT_{2x} =1.5 to 8.0 K. We see our range of climate parameterizations broadly encompasses the range of climate models. See Supplementary Note 1 for full references and further explanation.

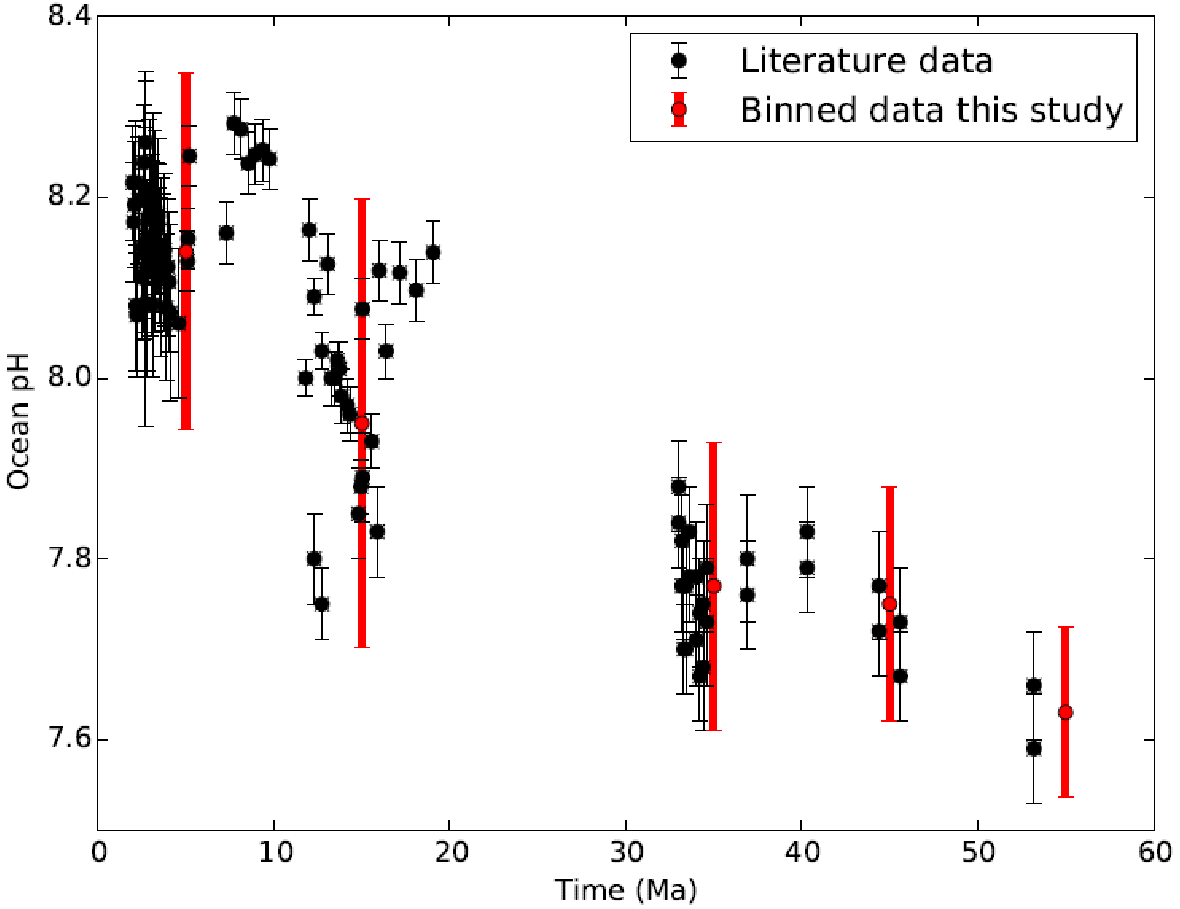

Supplementary Figure 10: Black squares show proxy estimates of ocean pH from the literature[4-9]. The red circles are the 10 Ma binned data used in this study. The uncertainty envelope for each binned data point is taken to be the range of proxy values within each 10 Ma bin.

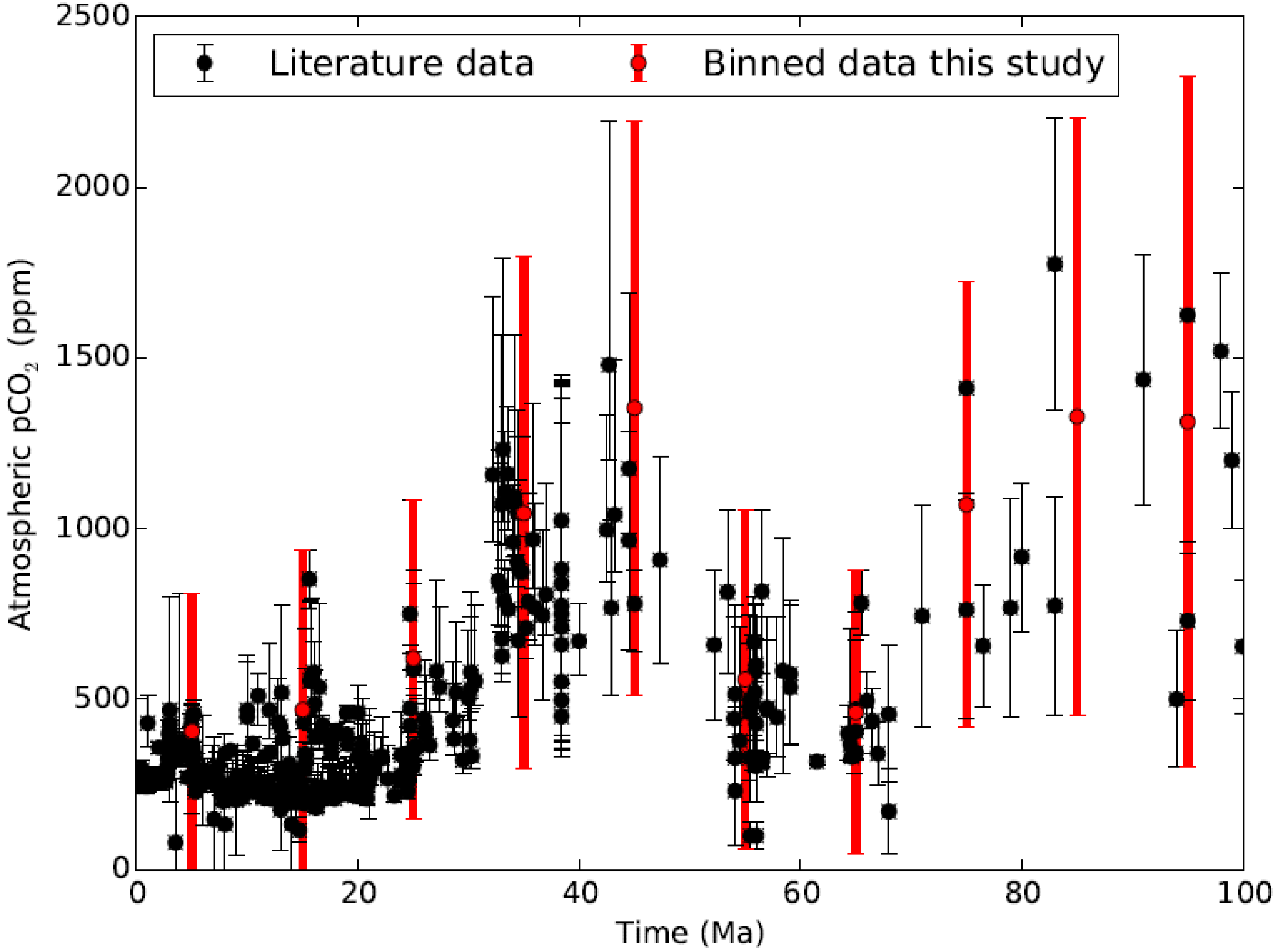

Supplementary Figure 11: Black squares show proxy estimates of atmospheric pCO_2 from the literature[10-16]. The red circles are the 10 Ma binned data used in this study. The uncertainty envelope for each binned data point is taken to be the range of proxy values within each 10 Ma bin.

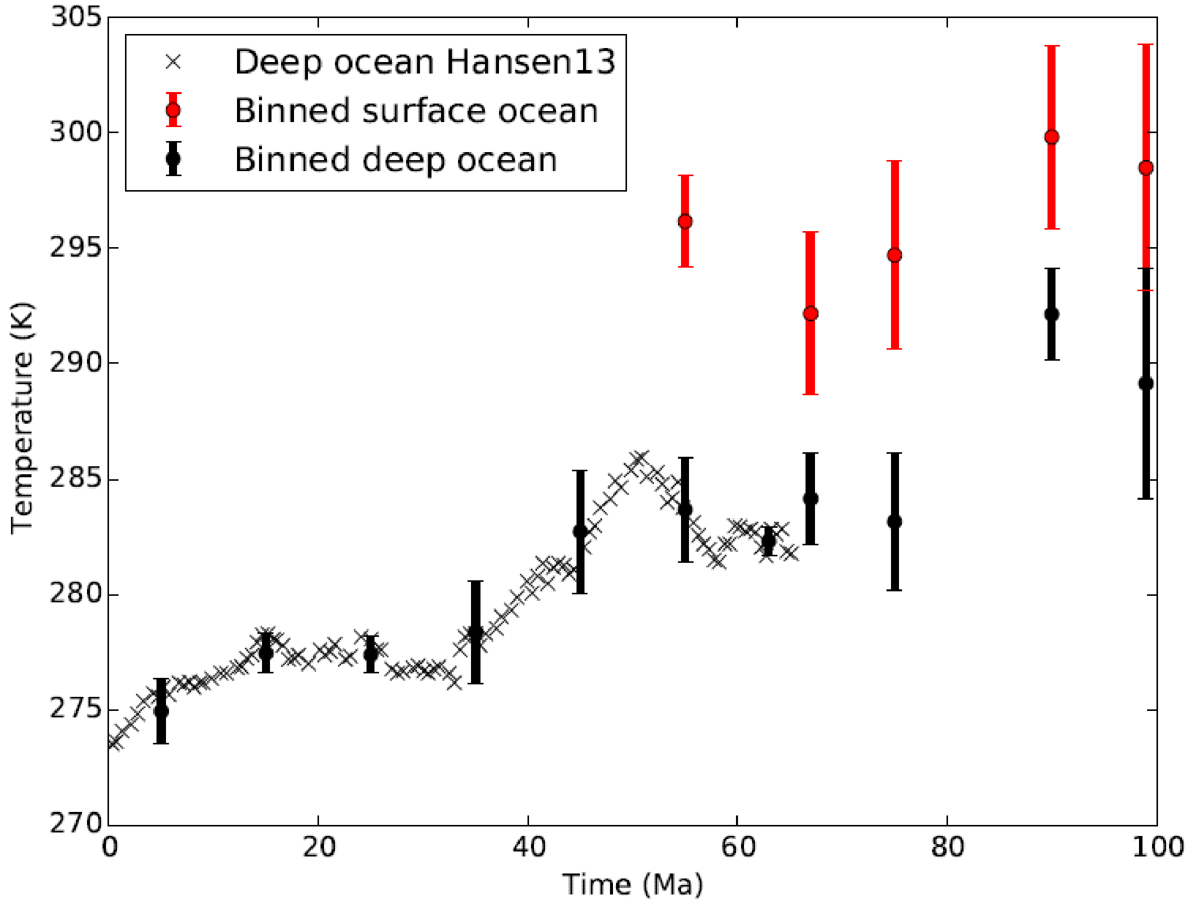

Supplementary Figure 12: Black and red circles show binned mean temperature values for the deep ocean and the surface, respectively. The proxy data used to construct these ranges is described in Supplementary Methods. Black crosses show Cenozoic deep ocean temperatures from Hansen *et al.*[17].

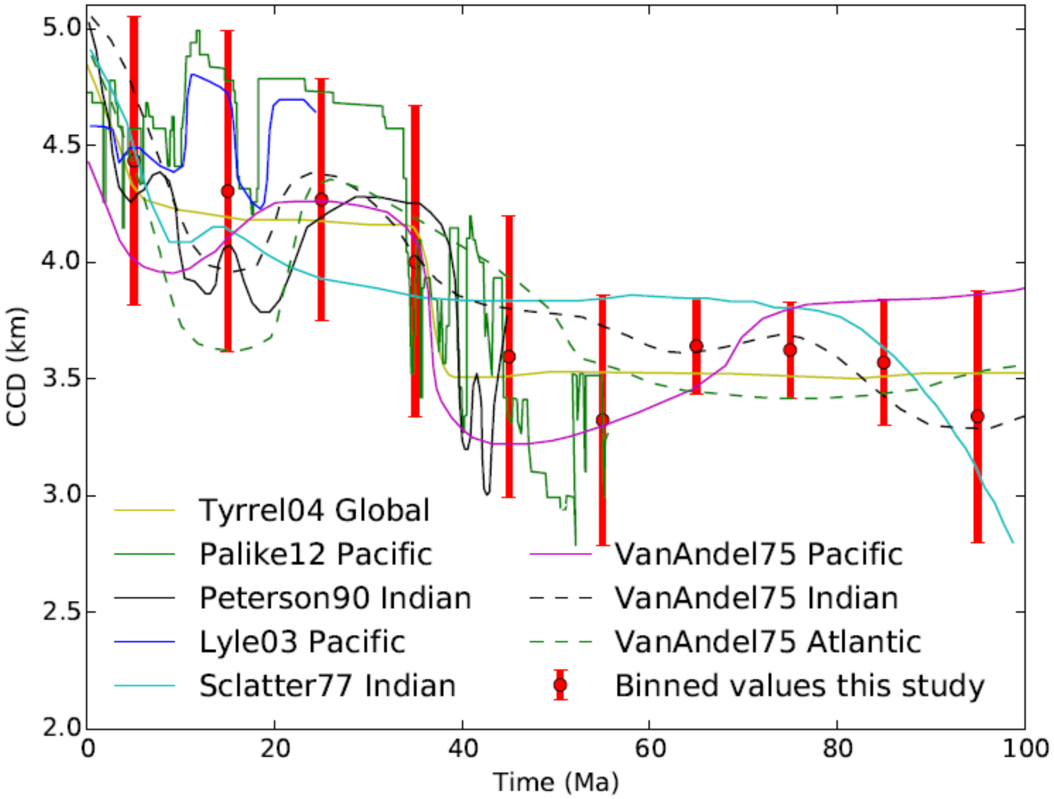

Supplementary Figure 13: Colored lines show estimates of Calcite Compensation Depth (CCD) for various ocean basins from the literature[3,18-22]. The red circles are the 10 Ma binned data used in this study. The uncertainty envelope for each binned data point is taken to be the range of reconstructions within that 10 Ma bin.

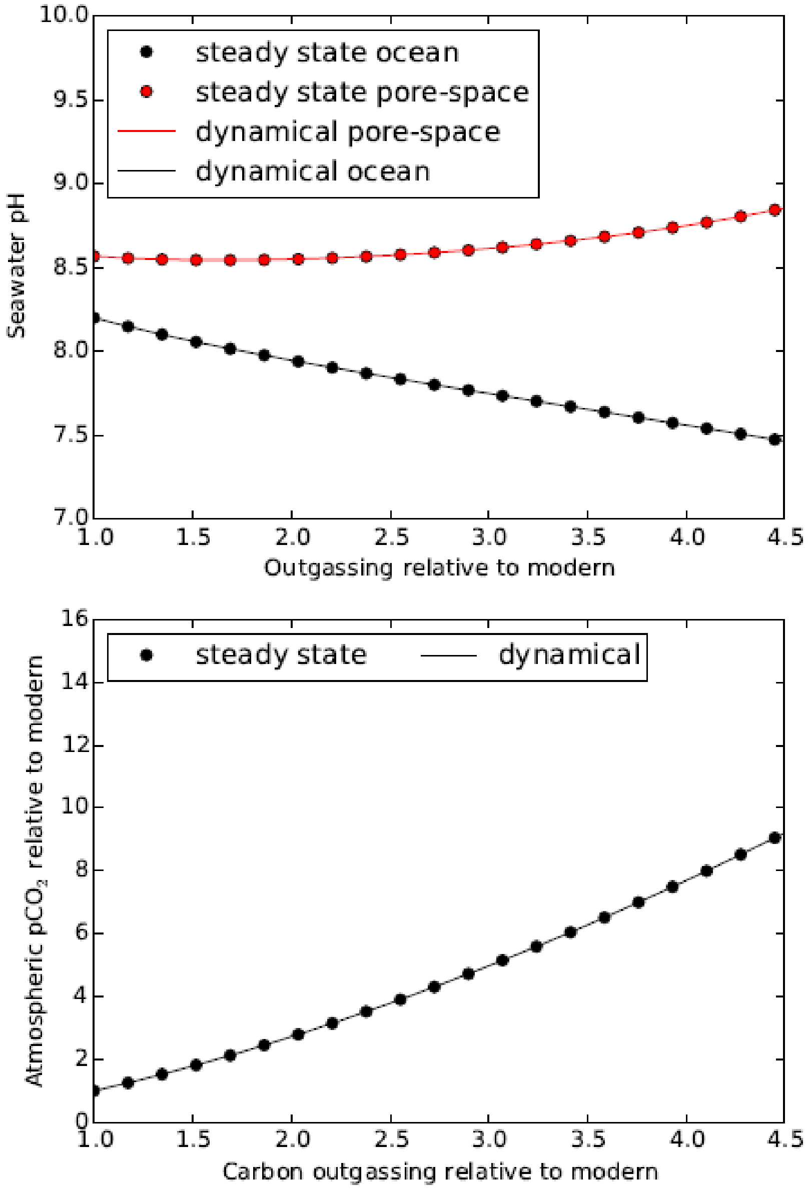

Supplementary Figure 14: Comparison of dynamical model (solid lines) and steady state calculations (circles). Supplementary Note 3 describes steady state calculations in full detail. There is excellent agreement between the two methods.

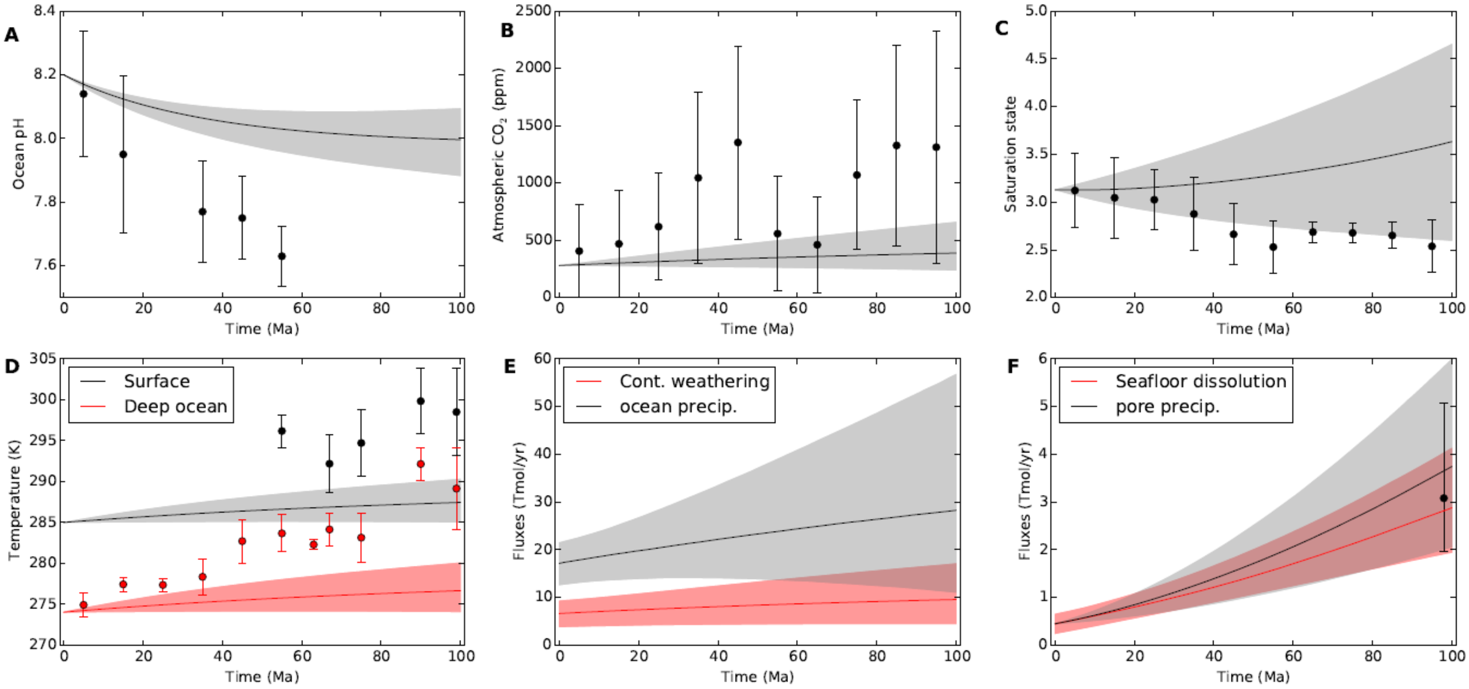

Supplementary Figure 15: An additional alkalinity source has been introduced in the pore space to account for K-feldspar formation (see Supplementary Note 4). Grey and red shaded regions represent the model output 90% confidence obtained from 10,000 forward model runs using the parameter ranges described in Table 1. The grey and red solid lines are the median model outputs. Black and red dots represent binned geochemical proxy data, and error bars denote the range of binned proxy estimates (see main text for references and explanation). The fit with pore-space precipitation is improved at the expense of a temperature and pH mismatch.

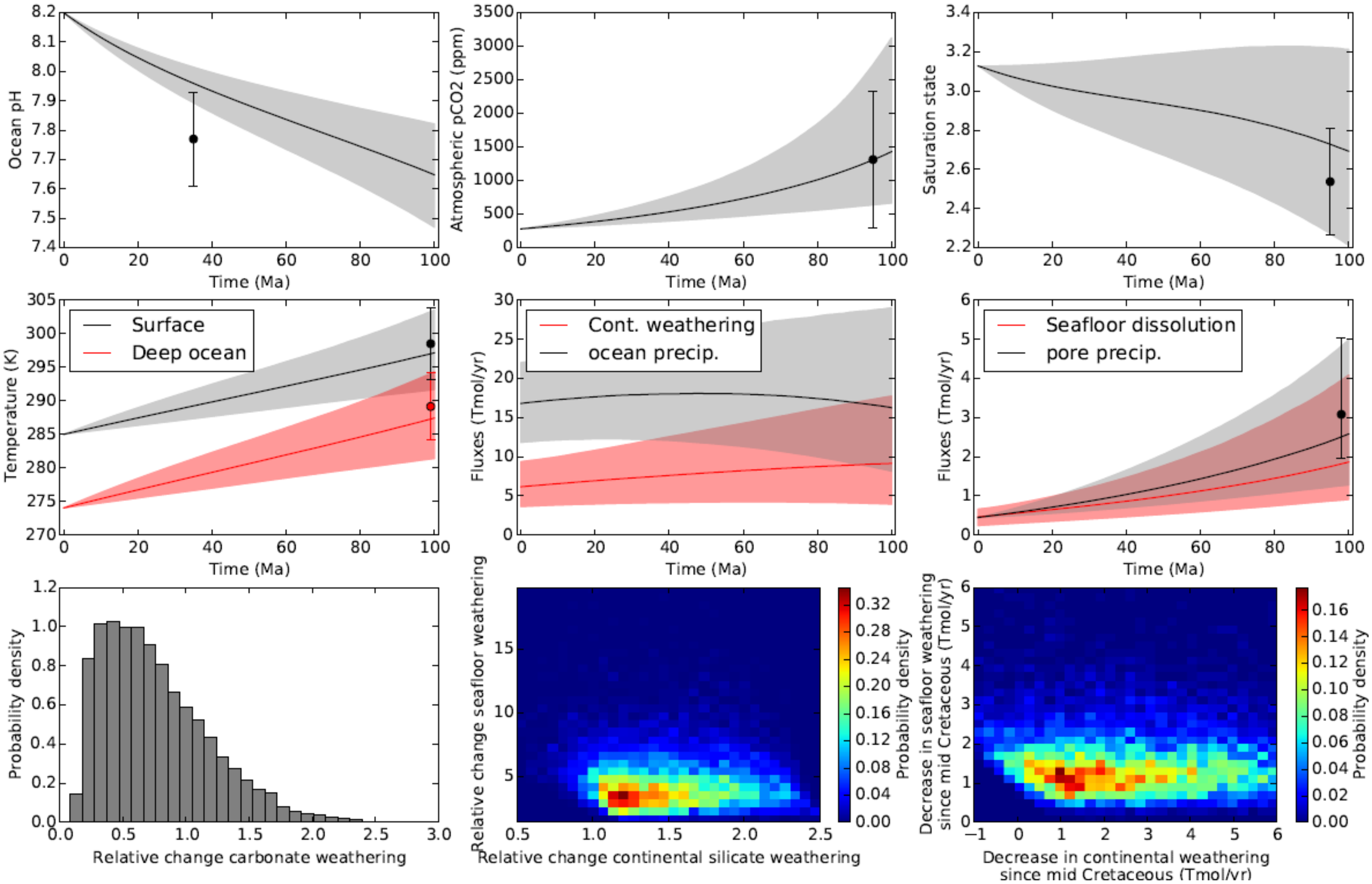

Supplementary Figure 16: Results of Bayesian inversion with limited data set. Each variable is fitted to a single mid Cretaceous data point (Cenozoic data point used for pH since no Cretaceous data exist). 1σ uncertainties in each data point are plotted, and shaded regions represent 95% credible intervals.

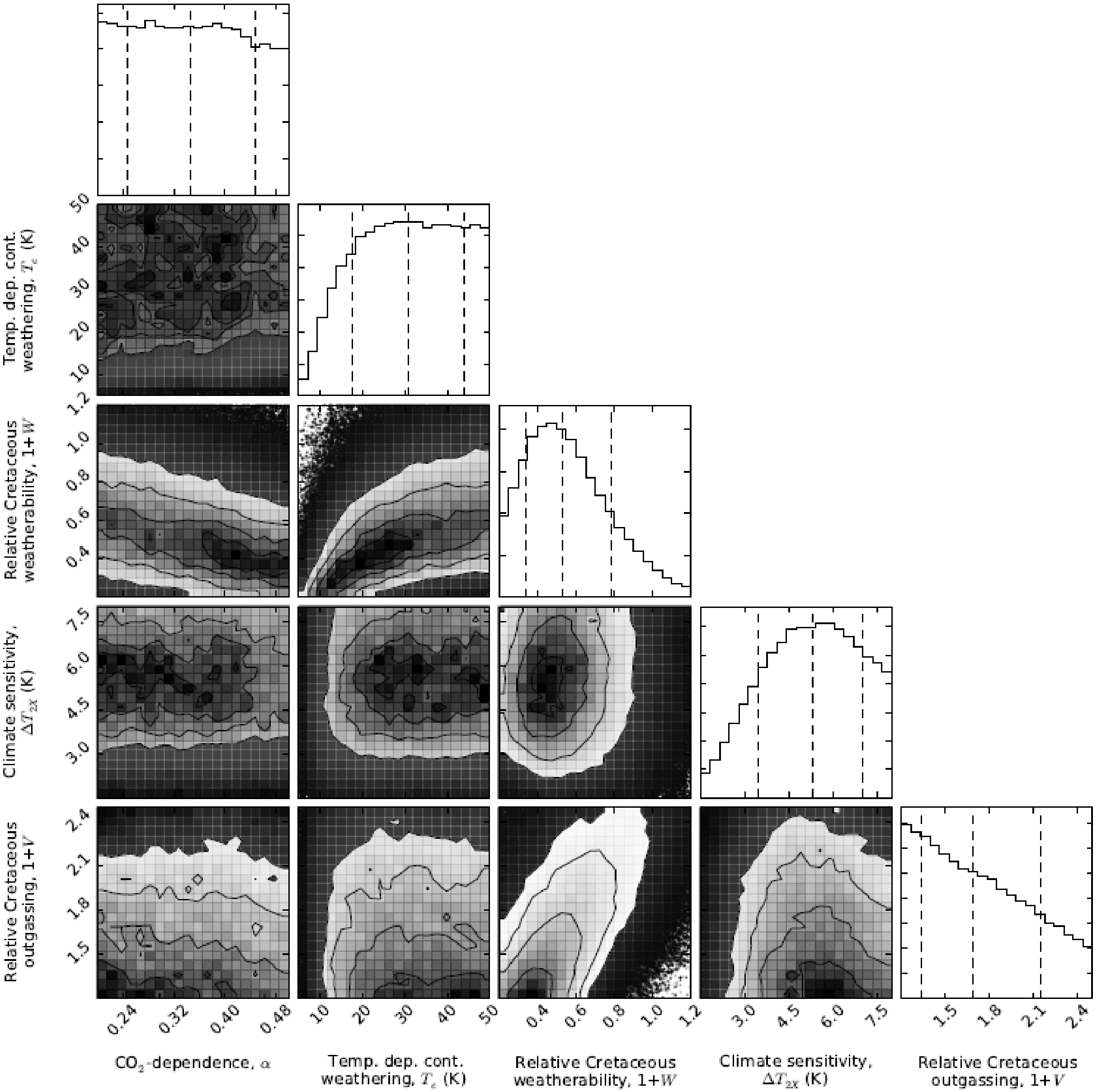

Supplementary Figure 17: Selected posterior probability distributions for the Bayesian inversion in Supplementary Fig. 16. Dotted lines represent the median value with 1σ error bars. The off-diagonal elements are joint probability distributions, which show how each pair of variables co-varies. Even when fitting a single mid Cretaceous data point for each variable – and thereby limiting the potential biases from underfitting and linearizing parameters - the general conclusions reported in the main text remain unchanged. Specifically, T_e is likely to be large, relative Cretaceous weatherability, 1+*W*, is approximately half modern weatherability, ΔT_{2x} is probably higher than fast feedback estimates.

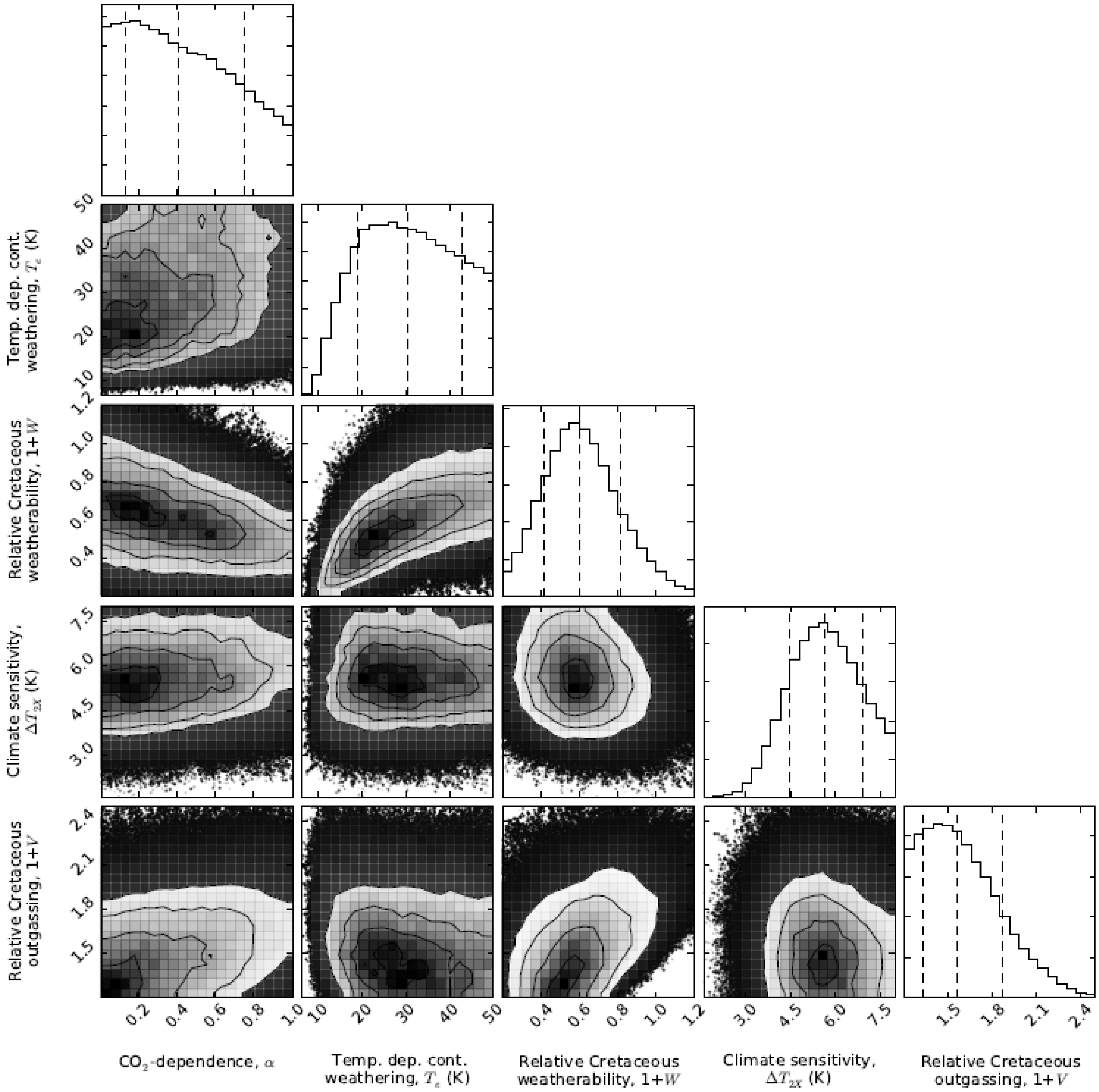

Supplementary Figure 18: Selected posterior probability distributions for a modified continental weathering function. The outer diagonal elements are the marginal distributions, and the off-diagonal elements are joint probability distributions, which show how each pair of variables co-varies. Dotted lines represent the median value with 1σ error bars. In this case the Michaelis-Menton law is adopted for the direct pCO_2 dependence of continental weathering (case 1). The 90% confidence intervals are $T_e = 14 - 48$ K, $1+W = 0.31 - 0.96$, and $\Delta T_{2x} = 3.8 - 7.6$ K.

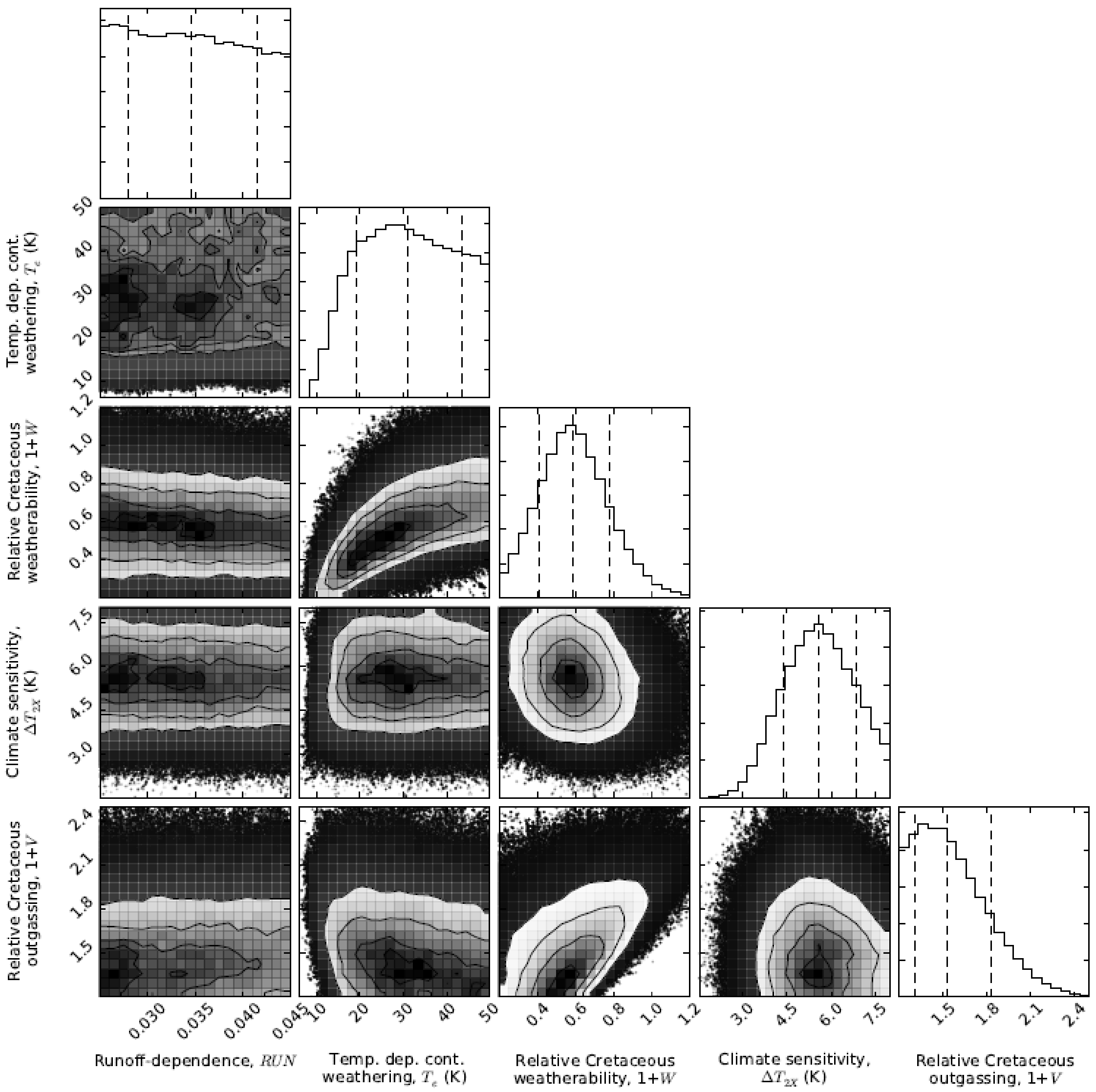

Supplementary Figure 19: Selected posterior probability distributions for a modified continental weathering function. The outer diagonal elements are the marginal distributions, and the off-diagonal elements are joint probability distributions, which show how each pair of variables co-varies. Dotted lines represent the median value with 1σ error bars. In this case the direct pCO_2 dependence of continental weathering is omitted and a linear runoff dependence is added (case 2). The 90% confidence intervals are $T_e = 14 - 48\,\mathrm{K}$, $1+W = 0.31 - 0.91$, and $\Delta T_{2x} = 3.7 - 7.5\,\mathrm{K}$.

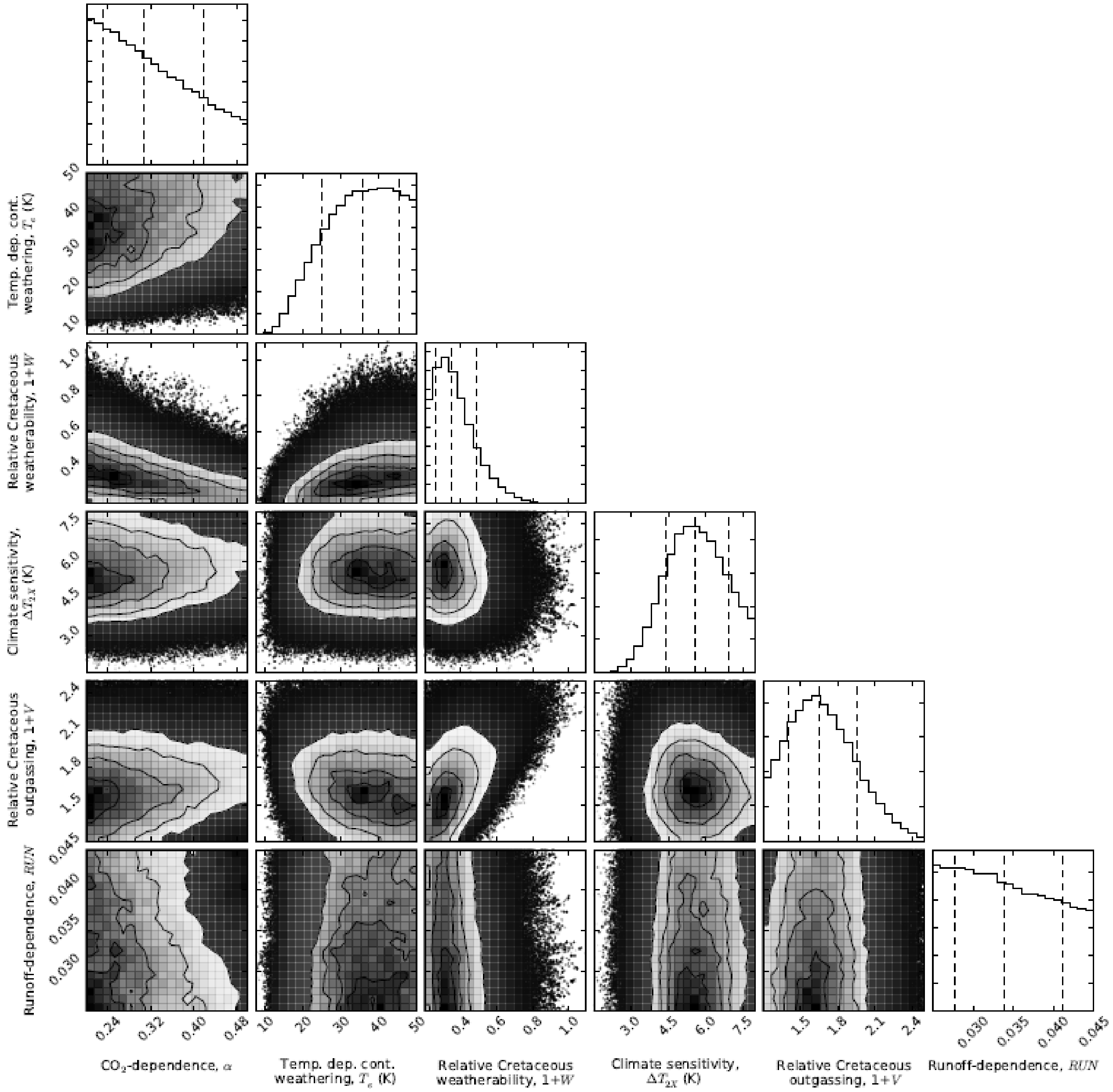

Supplementary Figure 20: Selected posterior probability distributions for a modified continental weathering function. The outer diagonal elements are the marginal distributions, and the off-diagonal elements are joint probability distributions, which show how each pair of variables co-varies. Dotted lines represent the median value with 1σ error bars. In this case the power law direct pCO_2 dependence of continental weathering is retained and a linear runoff dependence is added (case 3). The 90% confidence intervals are $T_e = 19 - 48\,\mathrm{K}$, $1+W = 0.22 - 0.60$, and $\Delta T_{2x} = 3.7 - 7.6\,\mathrm{K}$.

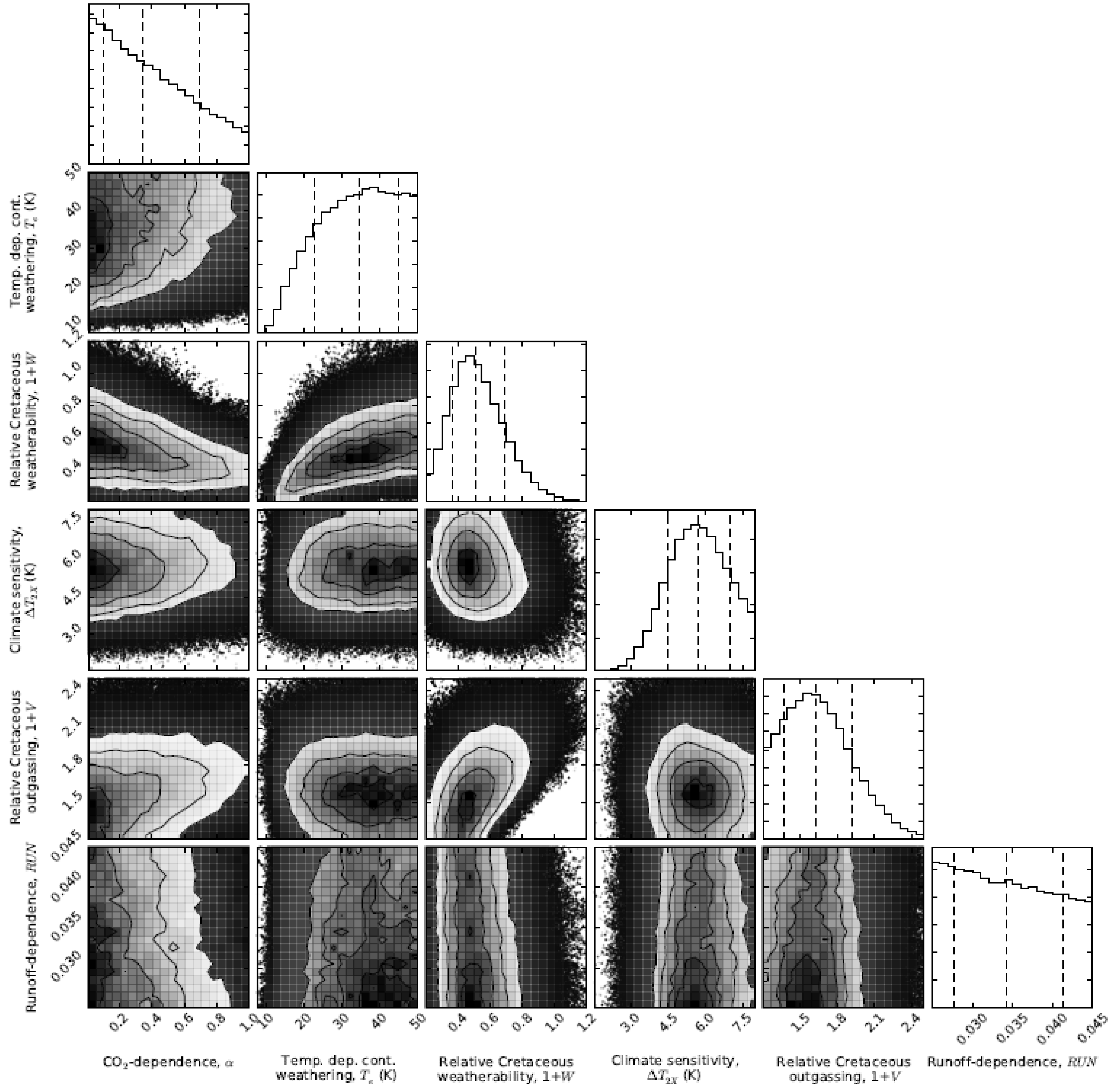

Supplementary Figure 21: Selected posterior probability distributions for a modified continental weathering function. The outer diagonal elements are the marginal distributions, and the off-diagonal elements are joint probability distributions, which show how each pair of variables co-varies. Dotted lines represent the median value with 1σ error bars. In this case the Michaelis-Menton law is adopted for the direct pCO_2 dependence of continental weathering and a linear runoff dependence is added (case 4). The 90% confidence intervals are $T_e = 17 - 48\,\mathrm{K}$, $1 + W = 0.28 - 0.83$, and $\Delta T_{2x} = 3.8 - 7.6\,\mathrm{K}$.

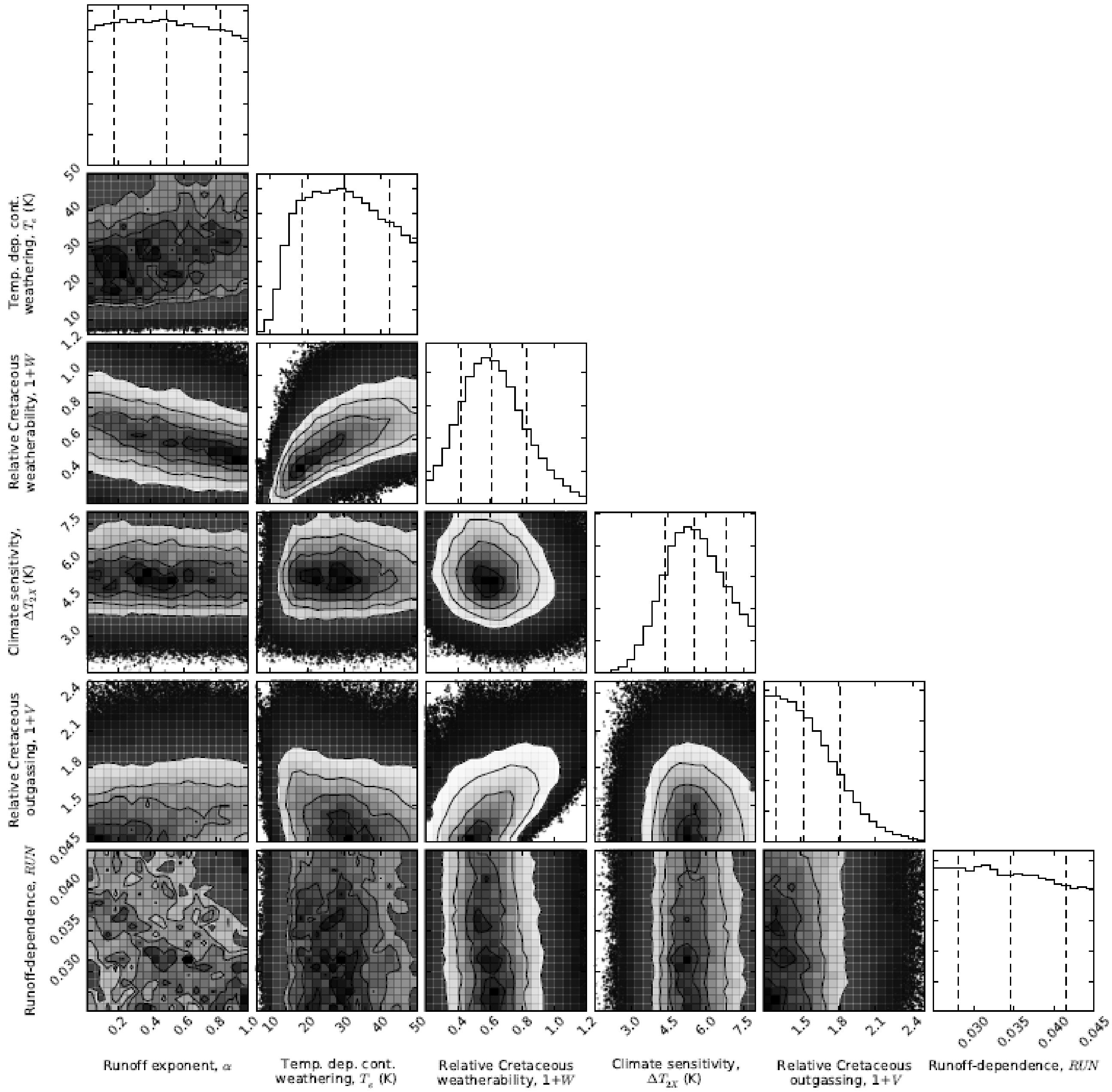

Supplementary Figure 22: Selected posterior probability distributions for a modified continental weathering function. The outer diagonal elements are the marginal distributions, and the off-diagonal elements are joint probability distributions, which show how each pair of variables co-varies. Dotted lines represent the median value with 1σ error bars. In this case the direct pCO_2 dependence of continental weathering is omitted and a linear runoff dependence is added with an additional runoff exponent (case 5). The 90% confidence intervals are $T_e = 14 - 47\ \mathrm{K}$, $1+W = 0.31 - 0.98$, and $\Delta T_{2x} = 3.7 - 7.5\ \mathrm{K}$.

Supplementary Note 1: Climate and deep-ocean temperature parameterizations

The Global Circulation Model (GCM) outputs used in Fig. 8 were taken from Li *et al.*[23], Stouffer and Manabe[24], and Danabasoglu and Gent[25]. These studies were chosen because they used fully coupled atmosphere-ocean GCMs with complete ocean circulation. Each GCM was run for thousands of years, which is sufficient time for the deep ocean to reach equilibrium.

Paleocene-Eocene Thermal Maximum (PETM) temperatures were sourced from Jones *et al.*[26]. The peak-PETM mean surface temperature, 27.3°C, was obtained by averaging all the reported temperatures in Table 1 of Jones *et al.*[26], and weighting each measurement by the cosine of its latitude. The pre-PETM mean surface temperature, 23.03°C, was obtained by subtracting the best-fit warming (4.3°C) from the peak-PETM temperature, although directly averaging pre-PETM measurements produced a similar result. Deep ocean pre-PETM and peak-PETM temperatures were estimated from Table 2 of Jones *et al.*[26]. Observed deep ocean temperatures were weighted by the areas of their respective ocean basins, and the pre-PETM and peak-PETM averages were found to be 12.1°C and 16.5°C, respectively. Error bars for PETM proxy temperatures were estimated as follows: McInerney and Wing[27] and Higgins and Schrag[28] estimate 5-8°C surface warming during the PETM, and so we adopted an error of 2°C in both the peak-PETM and pre-PETM surface temperatures.

Last Glacial Maximum (LGM) temperatures were estimated as follows. Clark *et al.*[29] gave the modern deep sea temperature as 1.3°C and the change since the LGM as 3.25±0.55°C. This implies the LGM deep ocean temperature was -2.2±0.55°C. Similarly, Schneider von Deimling *et al.*[30] estimated the mean surface cooling during the LGM to be 5.8±1.4°C, whereas Annan and Hargreaves[31] estimated the surface cooling to be 4±0.8°C. We averaged these estimates to obtain the mean surface cooling as 4.9±1.6°C, or equivalently the mean surface temperature 8.8±1.6°C.

Supplementary Fig. 9 shows the range of climate parameterizations considered in our model alongside selected GCM outputs and other simple climate models from the literature. The GCM outputs were taken from Li *et al.*[23], Stouffer and Manabe[24], Danabasoglu and Gent[25], Hansen *et al.*[32], and Meraner *et al.*[33]. The simple climate models were sourced from Abbot *et al.*[34], Walker *et al.*[35], Lenton[36], Pierrehumbert[37], and Kasting[38].

Supplementary Note 2: Climate model

The solar luminosity term in our climate model (equation (9)) was derived as follows. Doubling atmospheric CO_2 is equivalent to a 3.7 W m^{-2} radiative forcing[39]. Taking into account the geometry of insolation and assuming an albedo of 0.3, the solar luminosity decrease required to offset a 3.7 W m^{-2} radiative forcing is:

$$\left(4\times 3.7\ \mathrm{W/m^2}\right)\Big/\left(0.7\times 1366\ \mathrm{W/m^2}\right) = 0.01548 = 1.5\% \quad \text{(S1)}$$

Following Catling and Kasting[40] the evolution of relative solar luminosity, L, is approximated by:

$$L = \frac{1}{\left(1+0.4\left(t/4600\ \mathrm{Ma}\right)\right)} \tag{S2}$$

We seek the time, t (in Ma), that luminosity was 1.5% reduced relative to modern. Solving $0.9485 = 1\big/\left(1+0.4\left(t/4600\ \mathrm{Ma}\right)\right)$ yields $t = 181\ \mathrm{Ma}$ for the time at which the solar forcing was $-3.7\ \mathrm{W/m^2}$. If we were to assume that global temperatures respond to pCO_2 forcings in the same way as equivalent luminosity forcings then our climate equation would have the form (neglecting paleogeography):

$$\Delta T_{\mathrm{S}} = \Delta T_{2\mathrm{x}} \left(\frac{\ln\left(\mathrm{pCO_2}/\mathrm{pCO_2^{mod}}\right)}{\ln(2)} - \frac{t}{181\,Ma} \right) \tag{S3}$$

However, mean surfaces are slightly more sensitive to pCO_2 changes than to luminosity changes because the CO_2 forcing is more effective at high latitudes[41]. Hansen *et al.*[41] reported CO_2 forcings to be 1.26 times more effective than luminosity forcings and so we adopt this correction in our climate equation (neglecting paleogeography):

$$\Delta T_{\mathrm{S}} = \Delta T_{2\mathrm{x}} \left(\frac{\ln\left(\mathrm{pCO_2}/\mathrm{pCO_2^{mod}}\right)}{\ln(2)} - \frac{t}{1.26\times 181\,Ma} \right) \tag{S4}$$

The linear paleogeography term is described in the main text.

Supplementary Note 3: Validation of dynamical model with steady-state calculations

To validate our model we compared outputs to equivalent steady state calculations. To make the steady state calculations analytically tractable, it is necessary to simplify the dynamical equations somewhat. Many different cases were validated, and here we present one illustrative example. In this example the only sources/sinks are carbon outgassing, pore-space precipitation, and pore space basalt dissolution, i.e. no carbonate or silicate weathering. We also assume $s = 0$. Equation (6) can therefore be simplified to:

$$\begin{aligned}
\frac{dC_{\mathrm{O}}}{dt} &= -J\left(C_{\mathrm{O}} - C_{\mathrm{P}}\right)/M_{\mathrm{O}} + F_{\mathrm{out}}/M_{\mathrm{O}} \\
\frac{dA_{\mathrm{O}}}{dt} &= -J\left(A_{\mathrm{O}} - A_{\mathrm{P}}\right)/M_{\mathrm{O}} \\
\frac{dC_{\mathrm{P}}}{dt} &= J\left(C_{\mathrm{O}} - C_{\mathrm{P}}\right)/M_{\mathrm{P}} - P_{\mathrm{pore}}/M_{\mathrm{P}} \\
\frac{dA_{\mathrm{P}}}{dt} &= J\left(A_{\mathrm{O}} - A_{\mathrm{P}}\right)/M_{\mathrm{P}} + 2F_{\mathrm{diss}}/M_{\mathrm{P}} - 2P_{\mathrm{pore}}/M_{\mathrm{P}}
\end{aligned} \tag{S5}$$

The model was forced by increasing outgassing from modern to 4.5x modern levels over several billion years. Rather than specify calcium abundances, in this example we let $\left[\mathrm{Ca^{2+}}\right]$ evolve dynamically with alkalinity:

$$\left[\mathrm{Ca}^{2+}\right] = 0.5(\left[\mathrm{ALK}\right] - \left[\mathrm{ALK}\right]_{\mathrm{initial}}) + \left[\mathrm{Ca}^{2+}\right]_{\mathrm{initial}} \tag{S6}$$

Additionally, we ignore the pH dependence of basalt dissolution and assume dissolution is purely a function of temperature: $F_{\mathrm{diss}} = k_{\mathrm{T}} \exp\left(-E_{\mathrm{bas}} / RT_{\mathrm{pore}}\right)$. Selected model outputs from this dynamical calculation are plotted in Supplementary Fig. 14.

The same problem was also solved by computing successive steady states. Starting from the initial (modern) steady state for the ocean and pore space, outgassing was incrementally increased up to 4.5x modern levels, and the new steady state was found at each outgassing level. This calculation is described in full below. In the equations that follow, P-subscripts denote pore-space variables, and O-subscripts denote ocean variables.

Steady state in equations (S5) implies that $F_{\mathrm{diss}} = P_{\mathrm{pore}} = F_{\mathrm{out}}$. Because dissolution is a function of deep ocean temperature only, T_{D}, T_{S} and pCO_2 can be calculated from equations (9) and (12). Similarly, since P_{pore} is known, Ω_{P} can be calculated from equation (19). In other words the steady state conditions allow us to determine pCO_2 and Ω_{P} for any given level of outgassing, and from these two variables the complete carbon chemistry of the ocean and pore space can be solved simultaneously. By substituting equations (23) and (24) into equations (21) we obtain:

$$\begin{aligned}
\mathrm{ALK}_{\mathrm{P}} &= \left[\mathrm{CO}_3^{2-}\right]_{\mathrm{P}} \left(2 + \frac{\left[\mathrm{H}^+\right]_{\mathrm{P}}}{\mathrm{K}_2^*}\right) \\
\mathrm{DIC}_{\mathrm{P}} &= \left[\mathrm{CO}_3^{2-}\right]_{\mathrm{P}} \left(1 + \frac{\left[\mathrm{H}^+\right]_{\mathrm{P}}}{\mathrm{K}_2^*} + \frac{\left[\mathrm{H}^+\right]_{\mathrm{P}}^2}{\mathrm{K}_1^*\mathrm{K}_2^*}\right) \\
\therefore \mathrm{ALK}_{\mathrm{P}} - \mathrm{DIC}_{\mathrm{P}} &= \left[\mathrm{CO}_3^{2-}\right]_{\mathrm{P}} \left(1 - \frac{\left[\mathrm{H}^+\right]_{\mathrm{P}}^2}{\mathrm{K}_1^*\mathrm{K}_2^*}\right)
\end{aligned} \tag{S7}$$

Next, the first expression in equation (S7) can be rearranged and substituted into the third expression as follows:

$$\left[H^+\right]_P = K_2^*\left(\frac{ALK_P}{\left[CO_3^{2-}\right]_P} - 2\right)$$

$$\therefore \left[H^+\right]_P^2 = \left(K_2^*\right)^2\left(\frac{ALK_P - 2\left[CO_3^{2-}\right]_P}{\left[CO_3^{2-}\right]_P}\right)^2$$

$$\therefore ALK_P - DIC_P = \left[CO_3^{2-}\right]_P\left(1 - \frac{K_2^*}{K_1^*}\left(\frac{ALK_P - 2\left[CO_3^{2-}\right]_P}{\left[CO_3^{2-}\right]_P}\right)^2\right) \tag{S8}$$

$$= \left[CO_3^{2-}\right]_P - \frac{K_2^*}{K_1^*}\left(\frac{{ALK_P}^2 - 4\,ALK_P\left[CO_3^{2-}\right]_P + 4\left[CO_3^{2-}\right]_P^2}{\left[CO_3^{2-}\right]_P}\right)$$

This can then be rearranged to obtain a quadratic in $\left[CO_3^{2-}\right]_P$:

$$\left(ALK_P - DIC_P\right)\left[CO_3^{2-}\right]_P = \left[CO_3^{2-}\right]_P^2 - \frac{K_2^*}{K_1^*}\left({ALK_P}^2 - 4\,ALK_P\left[CO_3^{2-}\right]_P + 4\left[CO_3^{2-}\right]_P^2\right)$$

$$\therefore \left[CO_3^{2-}\right]_P^2\left(1 - 4\frac{K_2^*}{K_1^*}\right) + \left[CO_3^{2-}\right]_P\left(4\frac{K_2^*}{K_1^*}ALK_P + DIC_P - ALK_P\right) - \frac{K_2^*}{K_1^*}{ALK_P}^2 = 0 \tag{S9}$$

Denote the solution to this quadratic $\left[CO_3^{2-}\right]_P = X\left(ALK_P, DIC_P\right)$. The steady state condition also implies $DIC_P = DIC_O - F_{out}/J$, and so we can substitute this into the quadratic solution to obtain

$$\left[CO_3^{2-}\right]_P = X\left(ALK_P, DIC_O - F_{out}/J\right) \tag{S10}$$

Next, we use equations (23) and (21) to express $1/\left[H^+\right]_O$ in terms of pCO_2 and ALK_P:

$$\frac{K_1^*\left[CO_2\,aq\right]_O}{\left[H^+\right]_O} = \left[HCO_3^-\right]_O = \left(ALK_O - 2\left[CO_3^{2-}\right]_O\right) = \left(ALK_O - \frac{2K_1^*K_2^*\left[CO_2\,aq\right]_O}{\left[H^+\right]_O^2}\right)$$

$$\therefore \frac{2K_1^*K_2^*}{\left[H^+\right]_O^2} + \frac{K_1^*}{\left[H^+\right]_O} - \frac{ALK_P}{\left[CO_2\,aq\right]_O} = 0 \tag{S11}$$

$$\therefore \frac{1}{\left[H^+\right]_O} = -K_1^* + \sqrt{\left(K_1^*\right)^2 + \frac{4K_1^*K_2^*ALK_O}{\left[CO_2\,aq\right]_O}} = -K_1^* + \sqrt{\left(K_1^*\right)^2 + \frac{4K_1^*K_2^*ALK_P}{pCO_2 \times H_{CO_2}}}$$

In the last line, we make use of the steady state condition $ALK_O = ALK_P$ and equation (22). Next, we use equations (21) to (24) to express DIC_O in terms of $1/[H^+]_O$ and pCO_2 :

$$\begin{aligned} DIC_O &= \left[CO_3^{2-}\right]_O + \left[HCO_3^-\right]_O + \left[CO_2aq\right]_O \\ &= \left[CO_2aq\right]_O \left(\frac{K_1^*K_2^*}{\left[H^+\right]_O^2} + \frac{K_1^*}{\left[H^+\right]_O} + 1 \right) \\ &= pCO_2 \times H_{CO_2} \left(\frac{K_1^*K_2^*}{\left[H^+\right]_O^2} + \frac{K_1^*}{\left[H^+\right]_O} + 1 \right) \end{aligned} \tag{S12}$$

Equation (S11) can now be substituted into equation (S12), yielding DIC_O as a function of purely pCO_2 and ALK_P . This combined expression can be substituted into our quadratic solution (S10), which results in an expression for $\left[CO_3^{2-}\right]_P$ that is purely a function of ALK_P and pCO_2 :

$$\left[CO_3^{2-}\right]_P = X\left(ALK_P, DIC_O\left(pCO_2\right) - F_{out}/J\right) \tag{S13}$$

Finally, from equations (20) and (S6) we have the pore-space saturation state:

$$\begin{aligned} \Omega_P &= \frac{\left[Ca^{2+}\right]_P \left[CO_3^{2-}\right]_P}{K_{sp}} = \frac{\left(1/2\left([ALK]_P - [ALK]_{P\text{-initial}}\right) + \left[Ca^{2+}\right]_{P\text{-initial}}\right)\left[CO_3^{2-}\right]_P}{K_{sp}} \\ &= \frac{\left(1/2\left([ALK]_P - [ALK]_{P\text{-initial}}\right) + \left[Ca^{2+}\right]_{P\text{-initial}}\right) X\left(ALK_P, DIC_O\left(pCO_2\right) - F_{out}/J\right)}{K_{sp}} \end{aligned} \tag{S14}$$

In this expression, the only unknown is ALK_P (recall that Ω_P and pCO_2 are readily calculated from steady state conditions). It is therefore possible to solve this equation numerically to find ALK_P. Once $ALK_P = ALK_O$ is known, DIC_O can be calculated from equations (S12) and (S11), and the remaining carbon chemistry is trivially solved using equations (21) to (26) in the main text. The results from this steady state calculation are plotted in Supplementary Fig. 14 and compared to equivalent dynamical model outputs. They are in agreement, which implies that the numerical integration is working correctly, and that the carbon cycle is in quasi-steady state.

Supplementary Note 4: Modified models – K-feldspar uptake

A caveat on our results is that the assumed functional relationship for seafloor weathering (equation (13)) does not accommodate the influence of changing seawater chemistry on basalt dissolution other than pH changes and imposed [Ca^{2+}] variation. Despite variations in the

continental weathering source, seawater [K^+] concentration has remained remarkably constant over the Phanerozoic[42]. This could be explained by uptake in K-feldspar in the seafloor[43]. A seafloor buffer on [K^+] is potentially significant for the carbon cycle because K-uptake in K-feldspar formation releases [Ca^{2+}] with ~7:2 stoichiometric ratio (see below), and so enhanced continental supply of K^+ could result in alkalinity release and carbonate formation in the seafloor, thereby providing an additional negative feedback on pCO_2 [43].

The stoichiometry of K-feldspar formation implies that the addition of 2.5 wt% CO_2 to oceanic crust requires the addition of 0.75 wt% of K_2O [43]:

$$\begin{aligned}&2\mathrm{NaAlSi_3O_8}+7\mathrm{CaAl_2Si_2O_8}+2\mathrm{K^+}+8\mathrm{SiO_2}\\&+5\mathrm{CO_2}+2\mathrm{HCO_3^-}+5\mathrm{H_2O}\\&=2\mathrm{KAlSi_3O_8}+2\mathrm{NaAl_7Si_{11}O_{30}}\left(\mathrm{OH}\right)_6+7\mathrm{CaCO_3}\end{aligned} \tag{S15}$$

Varying K-feldspar uptake can be incorporated in our model with an additional alkalinity source in the pore-space. Cretaceous crust K_2O content is approximately 0.4 wt% greater than Cenozoic crust [43], which implies up to 1.3 wt% CO_2 added from K-feldspar formation. To crudely incorporate this ALK source into our model we modified equation (6) as follows:

$$\frac{dA_\mathrm{P}}{dt}=J\left(A_\mathrm{O}-A_\mathrm{P}\right)/M_\mathrm{P}+2F_\mathrm{diss}/M_\mathrm{P}-2P_\mathrm{pore}/M_\mathrm{P}+\frac{t\times1.3\times10^{12}\left(F_\mathrm{out}/F_\mathrm{out}^\mathrm{mod}\right)}{100\ Ma\times M_\mathrm{P}} \tag{S16}$$

For typical changes in crustal production, $F_\mathrm{out}/F_\mathrm{out}^\mathrm{mod}=1.5$, the alkalinity source due to K-feldspar formation will be ~2 Tmol eq yr^{-1} at 100 Ma and 0 Tmol eq yr^{-1} in the modern pore-space.

Including this alkalinity source does not affect our conclusions. For the base case (no weatherability change, *W*=0, and conventional temperature dependence of silicate weathering, $T_e=5-15\ \mathrm{K}$) the seafloor precipitation flux at 100 Ma is a better fit with data, however the fit with temperature and CO_2 is worsened. The combination of a strong silicate weathering feedback and a large seafloor sink due to K-feldspar formation draws down too much pCO_2 and makes the Cretaceous climate unreasonably cold (Supplementary Fig. 15). If either the temperature sensitivity of silicate weathering is weakened or a large weatherability increase is imposed, then the fit with temperature and CO_2 is marginal. The best fit is achieved by assuming both a weak climate sensitivity ($T_e=30-40\ \mathrm{K}$) and a large weatherability change (*W*=0.4-0.6), not shown. In short, a sizeable change in weatherability and/or a low temperature dependence of silicate weathering is still required to fit proxy data.

Supplementary Note 5: Modified models –Fitting the mid Cretaceous mean state

One potential criticism of our methodology is that by imposing linear trends on some variables in our model we do not fully capture shorter timescale fluctuations. By fitting binned time series data in this way we are arguably underfitting and potentially underestimating the true uncertainty in unknown variables. To test this we repeated the MCMC inversion but simplified the data to include only one mid Cretaceous data point for each variable. By only fitting the mid Cretaceous

endpoint the misfit from imposing linear trends is minimized. In this calculation we are no longer fitting a time series but rather fitting mean Cretaceous conditions with broad uncertainties.

The model outputs for this calculation are shown in supplementary figures 16 and 17. Although the uncertainties in fitted parameters are somewhat larger than the nominal case, key conclusions are unchanged: a large weatherability increase is probable, the temperature sensitivity of continental weathering is weak, and Earth system climate sensitivity is likely to be higher than fast-feedback estimates. A high Earth system climate sensitivity is supported by numerous paleoclimate studies[44-47].

Supplementary Note 6: Modified models – Different functional forms for continental weathering

The precise functional form for continental weathering is uncertain, and so we repeated our inverse analysis using five different functional forms for continental silicate and carbonate weathering to see how the choice of function affected the probability distributions for key parameters).

Case 1 (Table 1 and Fig. 6 main text): Replace power law pCO_2 dependence with Michaelis-Menton law[48,49]:

$$\begin{aligned} F_{\mathrm{sil}} &= \omega F_{\mathrm{sil}}^{\mathrm{mod}} \left(\frac{2R_{\mathrm{CO_2}}}{1+R_{\mathrm{CO_2}}} \right)^{\alpha} \exp\left(\Delta T_{\mathrm{S}} / T_e \right) \\ F_{\mathrm{carb}} &= \omega_{\mathrm{carb}} \omega F_{\mathrm{carb}}^{\mathrm{mod}} \left(\frac{2R_{\mathrm{CO_2}}}{1+R_{\mathrm{CO_2}}} \right)^{\alpha} \exp\left(\Delta T_{\mathrm{S}} / T_e \right) \end{aligned} \tag{S17}$$

This expression represents the effect of CO_2 fertilization on vascular land plants. The unknown exponent, α, represents the efficiency of CO_2 fertilization. We vary α across the full range from 0 to 1 in our inverse analysis such that it encompasses the endmember case where there is no direct pCO_2 dependence. For most of this range, the Michaelis-Menton law provides a weaker pCO_2 dependence than the original power law. For simplicity we adopt the same functional form for carbonate weathering as for silicate weathering, but recall the carbonate weathering function contains an additional free weatherability parameter to account for any differences with silicate weathering. Selected case 1 results are presented in the main text in Table 1 and Fig. 6.

Case 2: No direct pCO_2 dependence and linear runoff dependence. The direct pCO_2 dependence of silicate weathering adopted in the main text is uncertain and potentially overstated. Here, we omit any pCO_2 dependence and instead use the functional forms for silicate weathering adopted in field studies (e.g. [50,51]) with an Arrhenius temperature dependence and linear runoff dependence. We relate runoff to temperature using an expression from the COPSE model[52], $runoff = (1 + RUN \times \Delta T)^{0.65}$.

$$F_{\text{sil}} = \omega F_{\text{sil}}^{\text{mod}} \left(1 + RUN \times \Delta T_{\text{S}}\right)^{0.65} \exp\left(\Delta T_{\text{S}} / T_e\right)$$
$$F_{\text{carb}} = \omega_{\text{carb}} \omega F_{\text{carb}}^{\text{mod}} \left(1 + RUN \times \Delta T_{\text{S}}\right)^{0.65} \exp\left(\Delta T_{\text{S}} / T_e\right) \tag{S18}$$

Here, *RUN* is a proportionality constant that relates changes in surface temperature to changes in runoff. We adopt the range *RUN*=0.025-0.045 which spans glacial to greenhouse conditions[53].

Case 3: Power law direct pCO_2 dependence and linear runoff dependence.

$$F_{\text{sil}} = \omega F_{\text{sil}}^{\text{mod}} \left(R_{\text{CO}_2}\right)^{\alpha} \exp\left(\Delta T_{\text{S}} / T_e\right) \left(1 + RUN \times \Delta T_{\text{S}}\right)^{0.65}$$
$$F_{\text{carb}} = \omega_{\text{carb}} \omega F_{\text{carb}}^{\text{mod}} \left(R_{\text{CO}_2}\right)^{\alpha} \exp\left(\Delta T_{\text{S}} / T_e\right) \left(1 + RUN \times \Delta T_{\text{S}}\right)^{0.65} \tag{S19}$$

Here α ranges from 0.2 to 0.5 as in the main text, and *RUN* ranges from 0.025 to 0.045 as above.

Case 4: Michaelis-Menton law for pCO_2 dependence and linear runoff dependence.

$$F_{\text{sil}} = \omega F_{\text{sil}}^{\text{mod}} \left(\frac{2R_{\text{CO}_2}}{1 + R_{\text{CO}_2}}\right)^{\alpha} \exp\left(\Delta T_{\text{S}} / T_e\right) \left(1 + RUN \times \Delta T_{\text{S}}\right)^{0.65}$$
$$F_{\text{carb}} = \omega_{\text{carb}} \omega F_{\text{carb}}^{\text{mod}} \left(\frac{2R_{\text{CO}_2}}{1 + R_{\text{CO}_2}}\right)^{\alpha} \exp\left(\Delta T_{\text{S}} / T_e\right) \left(1 + RUN \times \Delta T_{\text{S}}\right)^{0.65} \tag{S20}$$

Here α=0-1 and *RUN*=0.025-0.045.

Case 5: No direct pCO_2 dependence and linear runoff dependence with unknown exponent. Rather than adopt the runoff expression adopted in the COPSE[52] model, we instead assume the runoff exponent is unknown and allow it to vary freely from 0 to 1. This allows for a wide range of runoff sensitivities to climate:

$$F_{\text{sil}} = \omega F_{\text{sil}}^{\text{mod}} \exp\left(\Delta T_{\text{S}} / T_e\right) \left(1 + RUN \times \Delta T_{\text{S}}\right)^{\alpha}$$
$$F_{\text{carb}} = \omega_{\text{carb}} \omega F_{\text{carb}}^{\text{mod}} \exp\left(\Delta T_{\text{S}} / T_e\right) \left(1 + RUN \times \Delta T_{\text{S}}\right)^{\alpha} \tag{S21}$$

Here α=0-1 is the runoff exponent and *RUN*=0.025-0.045 is the runoff sensitivity.

Supplementary figures 18-22 show the posterior distributions for key parameters for cases 1-5, respectively. We observe that the distribution for climate sensitivity is virtually unchanged by the choice of weathering function. This is unsurprising since climate sensitivity is controlled by the functional form of the climate equation and pCO_2 and temperature proxies. Similarly, weak temperature sensitivity of continental weathering (large T_e) is required in all five cases, consistent with our original analysis (T_e=17-48 K, 90% confidence). A slightly stronger temperature dependence is more probable for cases 1, 2 and 5 (T_e≈14-48 K, 90% confidence). This is expected because if the direct pCO_2 dependence of continental weathering is weak

(case 1, Michaelis-Menton law) or absent (cases 2 and 5), then some temperature sensitivity is required to ensure continental weathering can respond to changes in climate and fit proxies. In the cases with a stronger pCO_2 dependence (case 3) or an additional temperature dependence (case 4, runoff dependence), a weaker Arrhenius relationship (larger T_e) is more probable. Finally, a smaller increase in weatherability since 100 Ma is more probable in cases 1, 2, and 5 than in the original results. This can also be understood in terms of a weaker or absent direct pCO_2 dependence: without a pCO_2 dependence decreasing continental weathering since 100 Ma, the counterbalancing increase in weatherability does not need to be as large in order to fit proxies.

In summary, the choice of functional form for continental weathering does not change any of our qualitative conclusions, namely climate sensitivity is large, the temperature dependence of continental weathering is weak, and there has been a sizeable increase in weatherability since 100 Ma due to factors other than climate. However, our quantitative estimates of key variables – particularly the weatherability change – could be refined by a better mechanistic understanding of continental weathering on the global scale.

Supplementary Note 7: Silicate weathering and outgassing

From equation (2) we have $F_{\mathrm{sil}} \propto F_{\mathrm{sil}}^{\mathrm{mod}} \exp\left(\Delta T_{\mathrm{S}} / T_e\right)$. This can be rearranged to obtain $\Delta T_{\mathrm{S}} \propto T_e \ln\left(F_{\mathrm{sil}} / F_{\mathrm{sil}}^{\mathrm{mod}}\right)$, and so the change in surface temperature required to double the silicate weathering flux is given by $\Delta T_{\mathrm{S}} \propto T_e \ln(2)$. This expression and the 1σ values for T_e in Table 1 were used to compute the doubling temperatures reported in the abstract.

The outgassing reconstructions in Fig. 7 are sourced from Van Der Meer *et al.*[54] (blue), Vérard *et al.*[55] (red), Cogné and Humler[56] (aqua), Seton *et al.*[57] (purple), Hansen and Wallmann[58] (green), and Berner[48] (GEOCARB, yellow). These reconstructions were chosen because they rely on several independent lines of evidence: reconstructions of plate extent and plate motion based on field geology, paleogeography and paleomagentic data[55], seismic imaging of subducted plates to infer variations in subducted plate length over time[54], and reconstructions of seafloor age and depth[56,57]. As noted in the main text, the outlying Cogné and Humler[56] reconstruction is disputed[57,59].

Supplementary Methods

Ocean chemistry

The saturation state for calcite is calculated using the following expression from Pilson[60], p. 410-411:

$$\log\left(\mathrm{K_{sp}}\right)=\log\left(\mathrm{K_{sp}^{o}}\right)+\left(-0.77712+0.0028426T+178.34/T\right)35^{0.5}$$
$$-0.07711\times 35+0.0041249\times 35^{1.5} \qquad \text{(S22)}$$
$$where \quad \log\left(\mathrm{K_{sp}^{o}}\right)=-171.9065-0.077993T+\frac{2839.319}{T}+71.595\log\left(T\right)$$

Here, T is the temperature of the ocean or pore-space. A constant salinity of 35 parts per thousand is assumed in this expression. Note that we have not included a correction for changing Mg^{2+} abundances (e.g. ref [3]). Hain *et al.*[61] showed that including an Mg^{2+} correction factor introduced significant error to $\mathrm{K_{sp}}$. This is because the empirical correction factor is normally offset by changes in $\mathrm{K_2^*}$ (defined in equation (S23)). The most accurate approach, short of implementing the Pitzer equations, is to use the standard, fixed thermodynamic constants[61]. We omit the pressure dependence of the solubility product because pressure is not varying through time (multiplication by a constant would not change model outputs since fluxes are scaled to fit modern fluxes).

The Henry's law constant, $\mathrm{H_{CO_2}}$, and the first and second apparent dissociation constants of carbonic acid, $\mathrm{K_1^*}$ and $\mathrm{K_2^*}$ respectively, were calculated using the expressions in Pilson[60], p. 111:

$$\log\left(\mathrm{H_{CO_2}}\right)=\frac{9345.17}{T}-167.8108+23.3585\log\left(T\right)$$
$$+\left(.023517-2.3656\times10^{-4}T+4.7036\times10^{-7}T^{2}\right)\times 35 \qquad \text{(S23)}$$
$$p\mathrm{K_1^*}=17.788-0.073104T-0.0051087\times35+1.1463\times10^{-4}T^{2}$$
$$p\mathrm{K_2^*}=20.919-0.064209T-0.011887\times35+8.7313\times10^{-5}T^{2}$$

For simplicity and computational efficiency we used a constant T=291.15 K in equation (S23), which is the modern surface ocean temperature (surface oceans are warmer than the continents plus oceans average of 285 K). We conducted test model runs with large changes in T and found that it doesn't change observable parameters (pH, CO_2, carbon fluxes, saturation state) appreciably. Using temperature-dependent constants results in only moderate changes in alkalinity and carbonate speciation.

Derivation of equation (25)

From the definition of carbon abundance (Tmol C kg^{-1}) in the atmosphere-ocean box we have:

$$\begin{aligned} C &= \mathrm{DIC} + s \times \mathrm{pCO_2} \\ &= \left[\mathrm{CO_3^{2-}}\right] + \left[\mathrm{HCO_3^-}\right] + \left[\mathrm{CO_2aq}\right] + \frac{s\left[\mathrm{CO_2aq}\right]}{\mathrm{H_{CO_2}}} \\ &= \left[\mathrm{CO_3^{2-}}\right] + \frac{\left[\mathrm{H^+}\right]\left[\mathrm{CO_3^{2-}}\right]}{\mathrm{K_2^*}} + \frac{\left[\mathrm{H^+}\right]^2\left[\mathrm{CO_3^{2-}}\right]}{\mathrm{K_1^*K_2^*}} + \frac{s\left[\mathrm{H^+}\right]^2\left[\mathrm{CO_3^{2-}}\right]}{\mathrm{H_{CO_2}}\,\mathrm{K_1^*K_2^*}} \\ &= \left[\mathrm{CO_3^{2-}}\right]\left(1 + \frac{\left[\mathrm{H^+}\right]}{\mathrm{K_2^*}} + \frac{\left[\mathrm{H^+}\right]^2}{\mathrm{K_1^*K_2^*}}\left(1 + \frac{s}{\mathrm{H_{CO_2}}}\right)\right) \end{aligned} \tag{S24}$$

For the atmosphere-ocean reservoir, $s = n_{\mathrm{atm}} / M_{\mathrm{O}}$, where $n_{\mathrm{atm}} = 1.8 \times 10^{20}$ is the total number of moles in the atmosphere and M_{O} is the ocean mass in kg. For the pore-space $s = 0$ because the pore-space is not in contact with any gaseous reservoir of carbon.

Next, from the definition of alkalinity in the atmosphere-ocean box we have:

$$\begin{aligned} \mathrm{ALK} &= 2\left[\mathrm{CO_3^{2-}}\right] + \left[\mathrm{HCO_3^-}\right] \\ &= 2\left[\mathrm{CO_3^{2-}}\right] + \frac{\left[\mathrm{H^+}\right]\left[\mathrm{CO_3^{2-}}\right]}{\mathrm{K_2^*}} \\ &= \left[\mathrm{CO_3^{2-}}\right]\left(2 + \frac{\left[\mathrm{H^+}\right]}{\mathrm{K_2^*}}\right) \end{aligned} \tag{S25}$$

Combining equations (S24) and (S25) gives:

$$\begin{aligned} &\mathrm{ALK}\left(1 + \frac{\left[\mathrm{H^+}\right]}{\mathrm{K_2^*}} + \frac{\left[\mathrm{H^+}\right]^2}{\mathrm{K_1^*K_2^*}}\left(1 + \frac{s}{\mathrm{H_{CO_2}}}\right)\right) = C\left(2 + \frac{\left[\mathrm{H^+}\right]}{\mathrm{K_2^*}}\right) \\ &\frac{\mathrm{ALK}}{\mathrm{K_1^*K_2^*}}\left(1 + \frac{s}{\mathrm{H_{CO_2}}}\right)\left[\mathrm{H^+}\right]^2 + \left(\frac{\mathrm{ALK} - C}{\mathrm{K_2^*}}\right)\left[\mathrm{H^+}\right] + \mathrm{ALK} - 2C = 0 \end{aligned} \tag{S26}$$

This quadratic can be solved to find the $\left[\mathrm{H^+}\right]$ molality in either the ocean or the pore space.

Proxy records

This section describes the geochemical proxy data plotted in Fig. 2, 3, 4, 5, and supplementary figures 2, 15, and 16.

Ocean pH

Ocean pH estimates were taken from Anagnostou *et al.*[4], Tripati *et al.*[5], Foster *et al.*[6] and Bartoli *et al.*[7]. Data from Edgar *et al.*[8] and Pearson *et al.*[9] recalculated using the methodology of

Anagnostou *et al.*[4] were also used. Supplementary Fig. 10 shows all the data considered in this study and the 10 Ma binned data that were used in the forward modeling and inverse analyses described in the main text.

Ocean pH is typically inferred from boron isotope data. Boron speciation is pH dependent, and so ocean pH can be estimated from boron isotopes in carbonates, assuming the boron isotopic composition of seawater and the fractionation factor are known[62]. Anagnostou *et al.*[4] rigorously constrained Cenozoic ocean pH using a broad range of estimates for boron isotope vital effects and seawater isotopic composition. The resulting data form the basis of the pH dataset in this study. However, the uncertainty in these ocean pH estimates may still be underestimated if the computed values do not reflect a globally integrated signal. In the modern ocean, surface pH varies by at least 0.2 log units[63].

Atmospheric CO_2

Supplementary Fig. 11 shows the CO_2 proxy data considered in this analysis and the 10 Ma binned data that were used in the forward modeling and inverse analyses described in the main text. Cenozoic pCO_2 proxies were taken from Beerling and Royer[10]. This exhaustive compilation includes stomatal, paleosol, phytoplankton, liverwort, and boron-based estimates and represents a consensus reconstruction.

Cretaceous pCO_2 proxies were taken from Hong and Lee[11] (pedogenic carbonates), Franks *et al.*[12] (stomatal model), Fletcher *et al.*[13] (fossil bryophytes), Retallack[14] (stomatal index), Quan *et al.*[15] (stomatal index) and Barclay *et al.*[16] (stomatal index). Traditionally, isotopic methods such as those using pedogenic carbonates yield higher pCO_2 estimates than those from stomatal methods[64]. This discrepancy was partially resolved by Breecker *et al.*[65] who revaluated soil CO_2 concentrations downward yielding lower atmospheric pCO_2 estimates from pedogenic carbonates. However, Cretaceous pCO_2 estimates based on the Breecker *et al.*[65] methodology[11] remain significantly higher than recent contemporaneous stomatal estimates[12]. These recent stomatal estimates are based on a mechanistic model of leaf exchange rather than an empirical fit to stomatal indices and are arguably more accurate at high pCO_2[12]. However, the sensitivity of this model-based approach to parameter assumptions was highlighted by McElwain *et al.*[66], who concluded that Franks *et al.*[12] could have underestimated atmospheric pCO_2. In short, the discrepancy between stomatal and paleosol pCO_2 estimates remains unresolved. Consequently, we include data from both methods in Supplementary Fig. 11 and use the full range of proxy estimates to define the uncertainty in each 10 Ma interval.

Temperature

Paleocene-Eocene Thermal Maximum (PETM) temperatures are described in Supplementary Note 1. Cretaceous temperatures were estimated as follows. Deep ocean temperatures were taken from Fig. 3 in Huber *et al.*[67]. The four timespans reported in Huber *et al.*[67] are 66.5-68.5 Ma, 75.4-76.4 Ma, 92-94 Ma, 99-100 Ma, and for each interval deep ocean temperatures from benthic foraminifera were averaged across all latitudes to obtain 11°C, 10°C, 19°C, 16°C, respectively. The errors in these temperature estimates were taken to be 2°C, 3°C, 2°C, 5°C, respectively, based on the spread of data points in Fig. 3 in Huber *et al.*[67].

There is a broad range of estimates for Cretaceous mean surface temperatures in the literature. For example Hay and Floegel[68] presented a time series curve based on Frakes[69] and Frakes *et al.*[70] that gives the following surface averages: 22.6°C, 25.6°C, 30.7°C and 30.7°C for 65-70 Ma, 70-80 Ma, 85-95 Ma, and 95-105 Ma, respectively. However, a simple latitudinal average of temperatures in Frakes[69] (weighted by latitude) yields 15.5°C, 17.5°C, 22.7°C, and 20°C for the same set of intervals. Huber *et al.*[67] doesn't report mean surface temperatures, but a crude averaging of surface temperatures in their Fig. 4 suggests values of 17°C, 20°C, 27°C, and 28°C for the same time intervals. Simple latitude-weighted SST averages from Li and Keller[71] yields 18.5°C for 75 Ma and 15.4°C for 65 Ma. In this study we take a conservative approach by letting the smallest and largest reported surface temperatures for each timespan define our confidence interval, and use the midpoint as the best estimate. This yields the following Cretaceous mean surface temperatures: 19.0±3.5°C for 65-70 Ma, 21.6±4.1°C for 70-80 Ma, 26.7±4.0°C for 85-95 Ma, and 25.4±5.4°C for 95-105 Ma.

Cenozoic deep ocean temperatures were sourced from Hansen *et al.*[17]. We omit plotting Cenozoic surface temperatures for lack of a consensus time-series for globally averaged temperatures. Supplementary Fig. 12 summarizes all the temperature data used in this study, and shows the binned ranges used in the inverse analysis and forward modeling.

Saturation state

Supplementary Fig. 13 shows the variety of CCD reconstructions considered in this study[3,18-22]. The ranges of CCD reconstructions in each 10 Ma interval were used as the consensus CCD in this study. The CCD was converted to a saturation state using equation 4 in Jansen *et al.*[72]:

$$\Omega = k_{CCD} \exp\left(0.189\left(Z_{CCD} - 3.82\right)\right) \tag{S27}$$

Here k_{CCD} is chosen to match the modern saturation state in our model. Uncertainty in the Cretaceous saturation state is potentially underestimated because only a small number of independent studies sample this timespan.

Seafloor weathering

Estimates of the Cenozoic and Cretaceous seafloor precipitation fluxes were based on Gillis and Coogan[73]: The upper 300 m of Cenozoic-aged drill cores have CO_2 contents of 0.4±0.2 and 0.5±0.2 wt% (excluding young warm sites with extensive sediment cover). Thus the mean upper crust carbon content is 0.45±0.2 wt%. Gillis and Coogan[73] convert this to a carbon flux by assuming the modern crustal formation rate is 3 km^2 yr^{-1}, and then multiplying the resultant flux by a factor of 1.5 to correct for the lower crust contribution. Conveniently, the net effect of this conversion is equivalent to multiplying by (10^{12}/1.5)×1.5, and so wt% CO_2 can be easily converted to Tmol C yr^{-1} by multiplication by 10^{12}. Thus the initial value for the Cenozoic pore-space precipitation flux is 0.45 Tmol C yr^{-1} (Table 2). In contrast, Mills *et al.*[74] assumed a modern seafloor sink of 1.72 Tmol C yr^{-1} in their carbon cycle model. However, this flux was obtained from indiscriminately averaging the carbonate content in both Cenozoic and Mesozoic

oceanic crust. Overestimating the modern seafloor weathering flux will inflate the importance of the seafloor weathering sink at earlier times in Earth's history.

The upper 300 m of Cretaceous-aged drill cores have CO_2 contents of 2.4±0.7, 1.9±0.3, which gives a best-estimate of 2.35±0.75 wt% using the range method adopted for the other proxies. The cretaceous seafloor precipitation flux will therefore be $(2.35\pm0.75)\times(1+V)^{\beta}$ Tmol C yr^{-1}, where we have multiplied by the Cretaceous crustal production rate relative to modern. The error bars for the Cretaceous pore-space flux in Fig. 2, 3, 4, 5, and supplementary figures 2, 15, and 16 were obtained by sampling our assumed range for V and calculating the inferred range of fluxes. For the Bayesian analysis, the observed precipitation sink was calculated for each forward model call by multiplying by $(1+V)^{\beta}$.

Isotopic constraints

Isotopic records provide potentially powerful constraints on carbon cycle processes e.g. ref [75]. However, we have deliberately avoided using Sr, Os, or Li isotope records to constrain our model due to uncertainties in their respective interpretations. For example, the upward trend in $^{87}Sr/^{86}Sr$ over the Cenozoic has multiple interpretations (see main text), and the Li isotope record cannot be straightforwardly related to continental weathering fluxes[76-78].

Bayesian inversion

The log-likelihood function used in the Bayesian inversion is defined as follows[79]:

$$\log(L) = -\frac{1}{2}\left(\sum_k \sum_i \frac{\left(OBS_{i,k} - MOD_{i,k}\right)^2}{\sigma_{i,k}^2}\right) - \frac{1}{2}\sum_k \sum_i \log\left(2\pi\sigma_{i,k}^2\right) \quad \text{(S28)}$$

Here, $OBS_{i,k}$, $MOD_{i,k}$, and $\sigma_{i,k}$ are the observed (proxy) value, model value, and uncertainty in the observed value of the i-th data point of the k-th variable, respectively. The k summation is over the six variables for which we have proxy records: mean surface temperature, mean deep ocean temperature, atmospheric pCO_2, ocean saturation state, ocean pH, and pore-space precipitation flux. The i summation is over the binned data plotted in the time series figures in the main text (Fig. 2, 3, 4, 5, and supplementary figures 2, 15, and 16), and described above.

The Bayesian analysis was implemented using the 'emcee' package in Python[80] and posterior distribution figures were created using the 'corner' module in Python. The emcee package implements an affine-invariant MCMC ensemble sampler. We used 1000 walkers and 10,000 model steps – that is a total of 10 million forward model calls – to build posterior distributions for our parameters. After accounting for autocorrelation in the walkers, the effective sample size was ~100,000. The initial walker positions were randomized, and a 1000 step burn-in was discarded. Approximately three quarters of walkers have crossed the median parameter value by 1000 steps, indicating that this burn-in is adequate. The 95% credible intervals plotted in Fig. 5 are the 2.5th-97.5th percentile range in the distribution of walkers.

A small number of studies have applied Bayesian methods to carbon cycle models. For

example, Royer *et al.*[81] and Park and Royer[45] adopted GEOCARBSULF[82] as a forward model and pCO_2 proxies to constrain Earth system climate sensitivity. Because these studies used pCO_2 proxies for the entire Phanerozoic they could estimate climate sensitivity separately for glacial and non-glacial periods. However, their retrieval only used pCO_2 to constrain model parameters and did not make use of temperature, saturation state, pH, and seafloor carbonate proxies. Additionally, the retrieval in Park and Royer[45] is dependent on the detailed parameterizations within GEOCARBSULF. Although some parameters such as silicate weathering activation energy and biological modifiers on weatherability were allowed to vary in Park and Royer[45], assumptions about the carbon and strontium isotope records and continental land area through time are 'hard-wired' into GEOCARBSULF. In contrast, we have deliberately designed our forward model to be as general as possible: isotope records were not used to force our model, and outgassing, carbonate and silicate weatherability, and modern-day fluxes are all free parameters. Consequently, the conclusions from our Bayesian analysis are more robust to model assumptions.

Supplementary References

1 Volk, T. Feedbacks between weathering and atmospheric CO2 over the last 100 million years *Am. J. Sci* **287,** 763-779 (1987).

2 Walker, L. J., Wilkinson, B. H. & Ivany, L. C. Continental drift and Phanerozoic carbonate accumulation in shallow-shelf and deep-marine settings *The Journal of Geology* **110,** 75-87 (2002).

3 Tyrrell, T. & Zeebe, R. E. History of carbonate ion concentration over the last 100 million years *Geochimica et Cosmochimica Acta* **68,** 3521-3530 (2004).

4 Anagnostou, E., John, E. H., Edgar, K. M., Foster, G. L., Ridgwell, A., Inglis, G. N. *et al.* Changing atmospheric CO2 concentration was the primary driver of early Cenozoic climate *Nature* **533,** 380-384 (2016).

5 Tripati, A. K., Roberts, C. D. & Eagle, R. A. Coupling of CO2 and ice sheet stability over major climate transitions of the last 20 million years *science* **326,** 1394-1397 (2009).

6 Foster, G. L., Lear, C. H. & Rae, J. W. The evolution of pCO 2, ice volume and climate during the middle Miocene *Earth and Planetary Science Letters* **341,** 243-254 (2012).

7 Bartoli, G., Hönisch, B. & Zeebe, R. E. Atmospheric CO2 decline during the Pliocene intensification of Northern Hemisphere glaciations *Paleoceanography* **26,** (2011).

8 Edgar, K. M., Anagnostou, E., Pearson, P. N. & Foster, G. L. Assessing the impact of diagenesis on δ 11 B, δ 13 C, δ 18 O, Sr/Ca and B/Ca values in fossil planktic foraminiferal calcite *Geochimica et Cosmochimica Acta* **166,** 189-209 (2015).

9 Pearson, P. N., Foster, G. L. & Wade, B. S. Atmospheric carbon dioxide through the Eocene–Oligocene climate transition *Nature* **461,** 1110-1113 (2009).

10 Beerling, D. J. & Royer, D. L. Convergent cenozoic CO2 history *Nature Geoscience* **4,** 418-420 (2011).

11 Hong, S. K. & Lee, Y. I. Evaluation of atmospheric carbon dioxide concentrations during the Cretaceous *Earth and Planetary Science Letters* **327,** 23-28 (2012).

12 Franks, P. J., Royer, D. L., Beerling, D. J., Van de Water, P. K., Cantrill, D. J., Barbour, M. M. *et al.* New constraints on atmospheric CO2 concentration for the Phanerozoic *Geophysical Research Letters* **41,** 4685-4694 (2014).

13 Fletcher, B. J., Beerling, D. J., Brentnall, S. J. & Royer, D. L. Fossil bryophytes as recorders of ancient CO2 levels: experimental evidence and a Cretaceous case study *Global Biogeochemical Cycles* **19,** (2005).

14 Retallack, G. J. A 300-million-year record of atmospheric carbon dioxide from fossil plant cuticles *Nature* **411,** 287-290 (2001).

15 Quan, C., Sun, C., Sun, Y. & Sun, G. High resolution estimates of paleo-CO 2 levels through the Campanian (Late Cretaceous) based on Ginkgo cuticles *Cretaceous Research* **30,** 424-428 (2009).

16 Barclay, R. S., McElwain, J. C. & Sageman, B. B. Carbon sequestration activated by a volcanic CO2 pulse during Ocean Anoxic Event 2 *Nature Geoscience* **3,** 205-208 (2010).

17 Hansen, J., Sato, M., Russell, G. & Kharecha, P. Climate sensitivity, sea level and atmospheric carbon dioxide *Philosophical Transactions of the Royal Society of London A: Mathematical, Physical and Engineering Sciences* **371,** 20120294 (2013).

18 Pälike, H., Lyle, M. W., Nishi, H., Raffi, I., Ridgwell, A., Gamage, K. *et al.* A Cenozoic record of the equatorial Pacific carbonate compensation depth *Nature* **488,** 609-614 (2012).

19 Van Andel, T. H. Mesozoic/Cenozoic calcite compensation depth and the global distribution of calcareous sediments *Earth and Planetary Science Letters* **26,** 187-194 (1975).

20 Lyle, M. Neogene carbonate burial in the Pacific Ocean *Paleoceanography* **18,** (2003).

21 Sclater, J. G., Abbott, D. & Thiede, J. Paleobathymetry and sediments of the Indian Ocean *Indian Ocean Geology and Biostratigraphy* 25-59 (1977).

22 Peterson, L. C. & Backman, J. in *ODP, Texas A&M University, College Station; UK distributors, IPOD Committee, NERC, Swindon.*

23 Li, C., von Storch, J.-S. & Marotzke, J. Deep-ocean heat uptake and equilibrium climate response *Climate Dynamics* **40,** 1071-1086 (2013).

24 Stouffer, R. & Manabe, S. Equilibrium response of thermohaline circulation to large changes in atmospheric CO2 concentration *Climate Dynamics* **20,** 759-773 (2003).

25 Danabasoglu, G. & Gent, P. R. Equilibrium climate sensitivity: Is it accurate to use a slab ocean model? *Journal of Climate* **22,** 2494-2499 (2009).

26 Jones, T. D., Lunt, D. J., Schmidt, D. N., Ridgwell, A., Sluijs, A., Valdes, P. J. *et al.* Climate model and proxy data constraints on ocean warming across the Paleocene–Eocene Thermal Maximum *Earth-Science Reviews* **125,** 123-145 (2013).

27 McInerney, F. A. & Wing, S. L. The Paleocene-Eocene Thermal Maximum: A perturbation of carbon cycle, climate, and biosphere with implications for the future *Annual Review of Earth and Planetary Sciences* **39,** 489-516 (2011).

28 Higgins, J. A. & Schrag, D. P. Beyond methane: towards a theory for the Paleocene–Eocene thermal maximum *Earth and Planetary Science Letters* **245,** 523-537 (2006).

29 Clark, P. U., Dyke, A. S., Shakun, J. D., Carlson, A. E., Clark, J., Wohlfarth, B. *et al.* The last glacial maximum *science* **325,** 710-714 (2009).

30 Schneider von Deimling, T., Ganopolski, A., Held, H. & Rahmstorf, S. How cold was the last glacial maximum? *Geophysical Research Letters* **33,** (2006).

31 Annan, J. & Hargreaves, J. A new global reconstruction of temperature changes at the Last Glacial Maximum *Climate of the Past* **9,** 367-376 (2013).

32 Hansen, J., Sato, M., Ruedy, R., Nazarenko, L., Lacis, A., Schmidt, G. *et al.* Efficacy of climate forcings *Journal of Geophysical Research: Atmospheres* **110,** (2005).

33 Meraner, K., Mauritsen, T. & Voigt, A. Robust increase in equilibrium climate sensitivity under global warming *Geophysical Research Letters* **40,** 5944-5948 (2013).

34 Abbot, D. S., Cowan, N. B. & Ciesla, F. J. Indication of insensitivity of planetary weathering behavior and habitable zone to surface land fraction *The Astrophysical Journal* **756,** 178 (2012).

35 Walker, J. C., Hays, P. & Kasting, J. F. A negative feedback mechanism for the long-term stabilization of Earth's surface temperature *Journal of Geophysical Research: Oceans (1978–2012)* **86,** 9776-9782 (1981).

36 Lenton, T. M. Land and ocean carbon cycle feedback effects on global warming in a simple Earth system model *Tellus B* **52,** 1159-1188 (2000).

37 Pierrehumbert, R. T. *Principles of planetary climate*. (Cambridge University Press, 2010).

38 Kasting, J. Proterozoic climates: the effect of changing atmospheric carbon dioxide concentrations *The Proterozoic Biosphere* 165-168 (1992).

39 IPCC. Climate change 2001: the scientific basis. Contribution of working group I to the third assessment report of the intergovernmental panel on climate change. Report No. 1477-8696, (Intergovernmental Panel on Climate Change, Cambridge, UK, 2001).

40 Catling, D. C. & Kasting, J. *Atmospheric Evolution on Inhabited and Lifeless Worlds*. (Cambridge University Press, 2017).

41 Hansen, J., Sato, M. & Ruedy, R. Radiative forcing and climate response *Journal of Geophysical Research: Atmospheres* **102,** 6831-6864 (1997).

42 Horita, J., Zimmermann, H. & Holland, H. D. Chemical evolution of seawater during the Phanerozoic: Implications from the record of marine evaporites *Geochimica et Cosmochimica Acta* **66,** 3733-3756 (2002).

43 Coogan, L. A. & Gillis, K. M. Evidence that low-temperature oceanic hydrothermal systems play an important role in the silicate-carbonate weathering cycle and long-term climate regulation *Geochemistry, Geophysics, Geosystems* **14,** 1771-1786 (2013).
44 Royer, D., Pagani, M. & Beerling, D. Geobiological constraints on Earth system sensitivity to CO2 during the Cretaceous and Cenozoic *Geobiology* **10,** 298-310 (2012).
45 Park, J. & Royer, D. L. Geologic constraints on the glacial amplification of Phanerozoic climate sensitivity *American Journal of Science* **311,** 1-26 (2011).
46 Royer, D. L. Climate Sensitivity in the Geologic Past *Annual Review of Earth and Planetary Sciences* 277-293 (2016).
47 Hansen, J., Sato, M., Kharecha, P., Beerling, D., Berner, R., Masson-Delmotte, V. *et al.* Target Atmospheric CO2: Where Should Humanity Aim? *Open Atmospheric Science Journal* **2,** 217-231 (2008).
48 Berner, R. A. *The Phanerozoic carbon cycle: CO2 and O2*. (Oxford University Press, 2004).
49 Volk, T. Rise of angiosperms as a factor in long-term climatic cooling *Geology* **17,** 107-110 (1989).
50 Dessert, C., Dupré, B., Gaillardet, J., François, L. M. & Allegre, C. J. Basalt weathering laws and the impact of basalt weathering on the global carbon cycle *Chemical Geology* **202,** 257-273 (2003).
51 Oliva, P., Viers, J. & Dupré, B. Chemical weathering in granitic environments *Chemical Geology* **202,** 225-256 (2003).
52 Bergman, N. M., Lenton, T. M. & Watson, A. J. COPSE: a new model of biogeochemical cycling over Phanerozoic time *American Journal of Science* **304,** 397-437 (2004).
53 Berner, R. A. & Kothavala, Z. GEOCARB III: a revised model of atmospheric CO2 over Phanerozoic time *American Journal of Science* **301,** 182-204 (2001).
54 Van Der Meer, D. G., Zeebe, R. E., van Hinsbergen, D. J., Sluijs, A., Spakman, W. & Torsvik, T. H. Plate tectonic controls on atmospheric CO2 levels since the Triassic *Proceedings of the National Academy of Sciences* **111,** 4380-4385 (2014).
55 Vérard, C., Hochard, C., Baumgartner, P. O., Stampfli, G. M. & Liu, M. Geodynamic evolution of the Earth over the Phanerozoic: Plate tectonic activity and palaeoclimatic indicators *Journal of Palaeogeography* **4,** 167-188 (2015).
56 Cogné, J. P. & Humler, E. Trends and rhythms in global seafloor generation rate *Geochemistry, Geophysics, Geosystems* **7,** (2006).
57 Seton, M., Gaina, C., Müller, R. & Heine, C. Mid-Cretaceous seafloor spreading pulse: Fact or fiction? *Geology* **37,** 687-690 (2009).
58 Hansen, K. W. & Wallmann, K. Cretaceous and Cenozoic evolution of seawater composition, atmospheric O2 and CO2: a model perspective *American Journal of Science* **303,** 94-148 (2003).
59 Müller, R. D., Sdrolias, M., Gaina, C., Steinberger, B. & Heine, C. Long-term sea-level fluctuations driven by ocean basin dynamics *science* **319,** 1357-1362 (2008).
60 Pilson, M. E. *An Introduction to the Chemistry of the Sea*. (Prentice-Hall, Inc., 1998).
61 Hain, M. P., Sigman, D. M., Higgins, J. A. & Haug, G. H. The effects of secular calcium and magnesium concentration changes on the thermodynamics of seawater acid/base chemistry: Implications for Eocene and Cretaceous ocean carbon chemistry and buffering *Global Biogeochemical Cycles* **29,** 517-533 (2015).
62 Hemming, N. G. & Hönisch, B. Chapter Seventeen Boron Isotopes in Marine Carbonate Sediments and the pH of the Ocean *Developments in Marine Geology* **1,** 717-734 (2007).
63 Takahashi, T., Sutherland, S. C., Chipman, D. W., Goddard, J. G., Ho, C., Newberger, T. *et al.* Climatological distributions of pH, pCO 2, total CO 2, alkalinity, and CaCO 3 saturation in the

global surface ocean, and temporal changes at selected locations *Marine Chemistry* **164,** 95-125 (2014).
64 Wang, Y., Huang, C., Sun, B., Quan, C., Wu, J. & Lin, Z. Paleo-CO 2 variation trends and the Cretaceous greenhouse climate *Earth-Science Reviews* **129,** 136-147 (2014).
65 Breecker, D., Sharp, Z. & McFadden, L. Atmospheric CO2 concentrations during ancient greenhouse climates were similar to those predicted for AD 2100 *Proceedings of the National Academy of Sciences* **107,** 576-580 (2010).
66 McElwain, J. C., Montañez, I., White, J. D., Wilson, J. P. & Yiotis, C. Was atmospheric CO 2 capped at 1000ppm over the past 300 million years? *Palaeogeography, Palaeoclimatology, Palaeoecology* **441,** 653-658 (2016).
67 Huber, B. T., Norris, R. D. & MacLeod, K. G. Deep-sea paleotemperature record of extreme warmth during the Cretaceous *Geology* **30,** 123-126 (2002).
68 Hay, W. W. & Floegel, S. New thoughts about the Cretaceous climate and oceans *Earth-Science Reviews* **115,** 262-272 (2012).
69 Frakes, L. A. Estimating the global thermal state from Cretaceous sea surface and continental temperature data *SPECIAL PAPERS-GEOLOGICAL SOCIETY OF AMERICA* 49-58 (1999).
70 Frakes, L. A., Probst, J.-L. & Ludwig, W. Latitudinal distribution of paleotemperature on land and sea from early Cretaceous to middle Miocene *Sciences de la terre et des planètes (Comptes rendus de l'Académie des sciences)* **318,** 1209-1218 (1994).
71 Li, L. & Keller, G. Variability in Late Cretaceous climate and deep waters: evidence from stable isotopes *Marine Geology* **161,** 171-190 (1999).
72 Jansen, H., Zeebe, R. E. & Wolf-Gladrow, D. A. Modeling the dissolution of settling CaCO3 in the ocean *Global Biogeochemical Cycles* **16,** (2002).
73 Gillis, K. & Coogan, L. Secular variation in carbon uptake into the ocean crust *Earth and Planetary Science Letters* **302,** 385-392 (2011).
74 Mills, B., Daines, S. J. & Lenton, T. M. Changing tectonic controls on the long-term carbon cycle from Mesozoic to present *Geochemistry, Geophysics, Geosystems* **15,** 4866-4884 (2014).
75 Li, G. & Elderfield, H. Evolution of carbon cycle over the past 100 million years *Geochimica et Cosmochimica Acta* **103,** 11-25 (2013).
76 von Strandmann, P. A. P., Burton, K. W., Opfergelt, S., Eiríksdóttir, E. S., Murphy, M. J., Einarsson, A. *et al.* The effect of hydrothermal spring weathering processes and primary productivity on lithium isotopes: Lake Myvatn, Iceland *Chemical Geology* (2016).
77 von Strandmann, P. A. P. & Henderson, G. M. The Li isotope response to mountain uplift *Geology* **43,** 67-70 (2015).
78 Wanner, C., Sonnenthal, E. L. & Liu, X.-M. Seawater δ 7 Li: A direct proxy for global CO 2 consumption by continental silicate weathering? *Chemical Geology* **381,** 154-167 (2014).
79 MacKay, D. J. *Information theory, inference and learning algorithms*. (Cambridge university press, 2003).
80 Foreman-Mackey, D., Hogg, D. W., Lang, D. & Goodman, J. emcee: the MCMC hammer *Publications of the Astronomical Society of the Pacific* **125,** 306 (2013).
81 Royer, D. L., Berner, R. A. & Park, J. Climate sensitivity constrained by CO2 concentrations over the past 420 million years *Nature* **446,** 530-532 (2007).
82 Berner, R. A. GEOCARBSULF: a combined model for Phanerozoic atmospheric O 2 and CO 2 *Geochimica et Cosmochimica Acta* **70,** 5653-5664 (2006).
